# Cell-type-specific alternative splicing in the Arabidopsis germline

**DOI:** 10.1093/plphys/kiac574

**Published:** 2022-12-14

**Authors:** Chandra Shekhar Misra, António G G Sousa, Pedro M Barros, Anton Kermanov, Jörg D Becker

**Affiliations:** Instituto de Tecnologia Química e Biológica António Xavier, Universidade Nova de Lisboa (ITQB NOVA), 2780-157 Oeiras, Portugal; Instituto Gulbenkian de Ciência, 2780-156 Oeiras, Portugal; Instituto Gulbenkian de Ciência, 2780-156 Oeiras, Portugal; Instituto de Tecnologia Química e Biológica António Xavier, Universidade Nova de Lisboa (ITQB NOVA), 2780-157 Oeiras, Portugal; Instituto de Tecnologia Química e Biológica António Xavier, Universidade Nova de Lisboa (ITQB NOVA), 2780-157 Oeiras, Portugal; Instituto Gulbenkian de Ciência, 2780-156 Oeiras, Portugal; Instituto de Tecnologia Química e Biológica António Xavier, Universidade Nova de Lisboa (ITQB NOVA), 2780-157 Oeiras, Portugal; Instituto Gulbenkian de Ciência, 2780-156 Oeiras, Portugal

## Abstract

During sexual reproduction in flowering plants, the two haploid sperm cells (SCs) embedded within the cytoplasm of a growing pollen tube are carried to the embryo sac for double fertilization. Pollen development in flowering plants is a dynamic process that encompasses changes at transcriptome and epigenome levels. While the transcriptome of pollen and SCs in Arabidopsis (*Arabidopsis thaliana*) is well documented, previous analyses have mostly been based on gene-level expression. In-depth transcriptome analysis, particularly the extent of alternative splicing (AS) at the resolution of SC and vegetative nucleus (VN), is still lacking. Therefore, we performed RNA-seq analysis to generate a spliceome map of Arabidopsis SCs and VN isolated from mature pollen grains. Based on our de novo transcriptome assembly, we identified 58,039 transcripts, including 9,681 novel transcripts, of which 2,091 were expressed in SCs and 3,600 in VN. Four hundred and sixty-eight genes were regulated both at gene and splicing levels, with many having functions in mRNA splicing, chromatin modification, and protein localization. Moreover, a comparison with egg cell RNA-seq data uncovered sex-specific regulation of transcription and splicing factors. Our study provides insights into a gamete-specific AS landscape at unprecedented resolution.

## Teaser

Transcriptome analysis of sperm cells and vegetative nuclei isolated from mature pollen grains reveals the alternative splicing landscape in the male gametophyte of Arabidopsis thaliana.

## Introduction

Male gametophyte development in flowering plants starts with haploid microspores, which undergo two consecutive pollen mitotic divisions to form mature pollen grains. A mature pollen grain contains two sperm cells (SCs), embedded within the vegetative cell of the pollen grain. The vegetative nucleus (VN) drives pollen tube growth, delivering the two SCs to the female gametophyte for double fertilization (reviewed in Berger and Twell, 2011).

Transcriptomes from all stages of pollen development and of isolated SCs have been extensively studied in Arabidopsis (*Arabidopsis thaliana*) (Honys and Twell, 2004; Pina et al., 2005; Borges et al., 2008). These studies were complemented by transcriptome analyses of pollen and SCs from other flowering plants, such as rice (*Oryza sativa*) (Anderson et al., 2013), maize (*Zea mays*) (Chen et al., 2017), and tomato (*Solanum lycopersicum*) (Liu et al., 2018). It is worth noting that except for rice, there is no transcriptome data available for the VN to date. Initially, studies on gene expression of male gametophytes were performed based on microarrays, but RNA-seq is the most commonly used technology nowadays. Reduction in cost coupled with high throughput transcriptomic analysis through RNA-seq offers several advantages over traditionally used DNA microarrays and one of them is the detection of alternative splice variants that are difficult to detect using arrays.

Alternative splicing (AS) is a mechanism of gene expression, in which more than one unique mRNA species is generated from a single gene (Baralle and Giudice, 2017). This enables cells to generate a vast diversity of proteins necessary for the normal growth and development of an organism. AS plays an important role in plant growth and development, regulating the circadian clock, flowering, and stress responses (Howard et al., 2013; Reddy et al., 2013; Staiger and Brown, 2013). In humans, up to 94% of intron-containing genes are alternatively spliced (Wang et al., 2008). It is estimated that up to 60% of intron-containing genes in Arabidopsis undergo AS (Filichkin et al., 2010; Marquez et al., 2012). This number varies among plant species, with, for instance, ∼52% in soybean (*Glycine max*) (Shen et al., 2014), 40% in cotton (*Gossypium raimondii*) and maize (Li et al., 2014; Thatcher et al., 2014), and 33% in rice (Zhang et al., 2010). The most predominant AS event in humans is exon skipping, whereas in plants, intron retention is considered to be the most predominant form of AS event, a notion that has recently been contested (Martín et al., 2021). More and more studies now predict that intron retention could be overestimated in plants, and non-intron retention events, such as exon skipping, could be more prevalent than originally thought (Martín et al., 2021). For instance, an alternative donor was found to be the most predominant form of AS during sugarcane (*Saccharum* spp.) smut disease infection (Bedre et al., 2019) and in barley (*Hordeum vulgare*) meiocytes (Barakate et al., 2020). In addition, exon skipping was the most predominant form of splicing event in developing maize ears (Thatcher et al., 2016), implying that tissue-specific AS is more diverse than previously considered.

The advent of deep sequencing has revealed in an unbiased manner how AS is coordinated during plant growth and development, and complementary studies aiming at understanding the functions of AS at a global level are also being carried out. For instance, genome-wide analysis of pollen-specific AS in Arabidopsis identified 30 genes to be differentially spliced between leaf and pollen (Loraine et al., 2013). One of these genes, *AtU2AF65a* (U2 small nuclear ribonucleoprotein auxiliary factor 65), was later characterized to have a role in regulating flowering time while displaying redundant roles in pollen tube growth together with its other isoform, *AtU2AF65b* (Park et al., 2019). More recently, genome-wide AS analysis was performed on five stages of microgametogenesis in field mustard (*Brassica rapa*), from pollen mother cell (PMC) to mature pollen grain (Golicz et al., 2021). The authors found a significant increase in intron retention during the meiotic stage transition from PMC to tetrad stage, with many of the genes having intron-retained isoforms resulting in nonfunctional proteins. Moreover, these isoforms were enriched in functions related to mRNA transport and ribosome biogenesis, indicating their possible role in regulating the rate of translation and maintaining the architecture of the nuclear envelope during meiosis (Golicz et al., 2021).

Apart from genome-wide analysis, currently, very few studies provide direct evidence to support a role of AS in plant reproduction. Only recently, the functions of some splicing factors have started to be uncovered, particularly those that could be involved in plant reproduction. For instance, in an Arabidopsis sr45 (serine/arginine-rich 45) mutant, pollen germinated earlier than that of the wild-type. AS analysis of the sr45 mutant showed that AtVLN1 (Arabidopsis VILLIN1), an actin-binding protein involved in pollen tube germination and growth, was affected. The expression level of the AtVLN1 full-length transcript was increased, while the transcript that produced a truncated protein decreased (Cao et al., 2013). These results indicate that SR45 might alter F-actin dynamics by directly or indirectly affecting AS of AtVLN1 and some other actin-binding proteins (Cao et al., 2013). The spliceosome subunit, PRP8 (Pre-mRNA PROCESSING factor 8), encoded by two paralogs, *PRP8A* and *PRP8B*, was found to be necessary for ovule competence for pollen tube attraction and potentially acted as an upstream regulator of pollen tube navigation toward ovules (Kulichová et al., 2020). The authors did not find any striking defect in prp8a prp8b double mutants that could be associated with male or female reproduction but proposed that these splicing factors may potentially facilitate male–female communication, an area of active research in the field of plant reproduction.

AS can occur either transcriptionally or cotranscriptionally, regulating development at specific time points, under stress conditions, or exerting a tissue-specific response. Genome-wide analysis of AS in different tissues in humans is well documented (Xu et al., 2002; Yeo et al., 2004; Pan et al., 2008), whereas in plants analysis of AS is limited to stress responses (Mandadi and Scholthof, 2015; Keller et al., 2017; Calixto et al., 2018; Huang et al., 2020), while tissue-specific AS analysis is limited (Martín et al., 2021).

In this study, we have used RNA-seq to study the transcriptome of SCs and VN isolated from Arabidopsis pollen grains. While the transcriptome of Arabidopsis SCs has been available for more than a decade (Borges et al., 2008), in this study we analyze the transcriptome of the VN in Arabidopsis. More importantly, we used our RNA-seq data to uncover genome-wide AS in Arabidopsis SCs and VN. Combining our data with a published dataset for Arabidopsis egg cells (ECs; Zhao et al., 2019) helped to uncover the genome-wide splicing landscape in gametes, thereby expanding the repertoire of genes involved in plant reproduction and their possible regulation through AS.

## Results

### The transcriptome of SCs and VN

**Figure 1:**
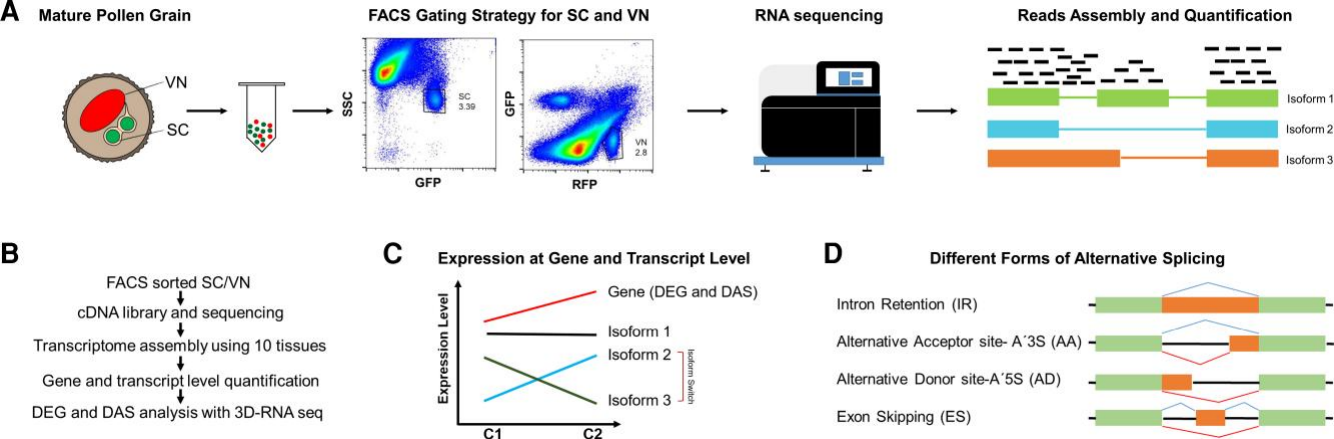
Flow diagram summarizing steps for the analysis of genome-wide AS in Arabidopsis SC and VN. A, Scheme showing FACS sorting strategy of GFP positive (MGH3p::MGH3-eGFP) SCs and RFP positive (ACT11p::H2B-mRFP) VN (Borges et al., 2008), followed by RNA-seq library preparation, sequencing, read assembly, and quantification. B, Flow chart depicting pipeline for analysis of AS. C, Example of a gene undergoing differential expression at the gene level or AS level. D, Different forms of AS events. SSC, side scatter.

We performed RNA-seq on SCs and VN isolated from mature pollen grains by FACS, aiming at an average of 30 million 150 bp paired-end reads per sample. A summary of FACS sorting, sequencing, and data analysis is shown in Figure 1.

**Figure 2:**
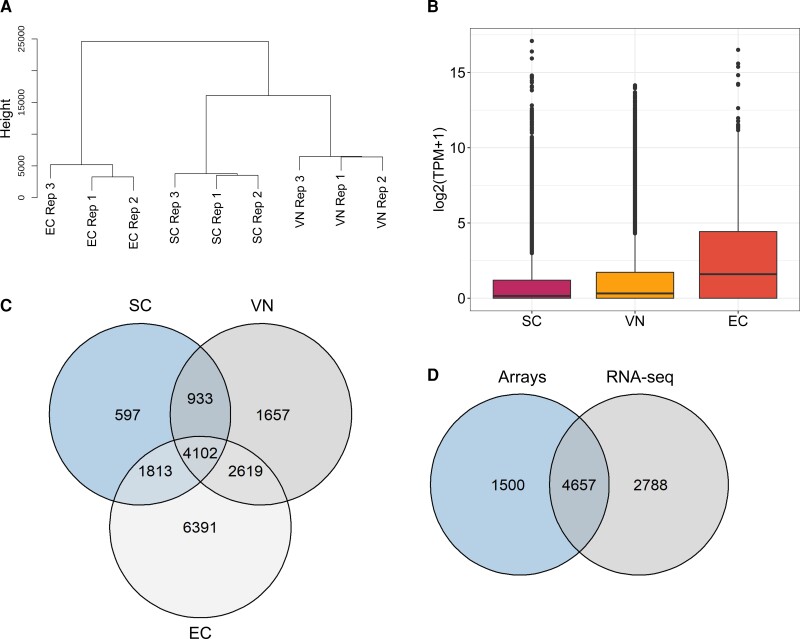
Transcriptome of Arabidopsis SC, VN, and EC. A, Hierarchical clustering of the three different cell types. B, Box plot representation of gene expression in SC, VN, and EC. C, Venn diagram showing the overlap between expressed genes between the three cell types at average TPM ≥ 1. D, Venn diagram showing overlap of expressed genes in SCs as detected by microarrays (Borges et al., 2008) and RNA-seq.

We also used published datasets from ECs (Zhao et al., 2019) to compare the cell-specific transcriptome profiles of the two germ cell types. To understand how the transcriptome of SCs and VN differed and to visualize overall differences between the male and female germline, we performed hierarchical clustering using all three sample types. The analysis revealed that the transcriptome of male and female germ cells clustered separately, whereas SCs and VN are clustered together; an observation that can be explained by SCs and VN deriving from the same precursor, the microspore (Figure 2A).

Our data suggest that the transcriptome diversity in SCs was reduced when compared to both VN and ECs (Figure 2B). The number of expressed genes in the three cell types (average expression transcripts per million [TPM] ≥ 1) ranged from 7,445 in SCs, over 9,310 in VN to 14,925 in ECs (Figure 2C) (Supplemental Table 1). Considerable overlap in the gene expression profiles was observed between the three cell types, but each of them also expressed transcripts unique to them (597 in SC, 1,657 in VN, and 6,391 in EC, respectively), indicating that these transcripts, in particular, may encode proteins performing functions specific to the respective cell type (Figure 2C).

The transcriptome of Arabidopsis gametes has been known for almost a decade (Borges et al., 2008; Wuest et al., 2010). However, due to limitations posed by the microarray platforms used in these studies, the number of genes that are expressed in SCs and EC were likely underestimated. A comparison of our RNA-seq and microarray data (Borges et al., 2008) revealed that 1,288 genes represented as probes on the arrays, but called absent in our array study, could be detected as expressed in our RNA-seq data (Figure 2D). In addition, RNA-seq helped to uncover the expression of an additional 2,788 genes for which there were no probes on the arrays (Figure 2D), 729 genes of which with high expression (TPM > 10) in SCs, including the well-known SC-specific transcription factor (TF) *DUO1* (*DUO POLLEN 1*) (Rotman et al., 2005; Brownfield et al., 2009a, 2009b). Interestingly, our results also show some genes that are exclusively detected as sperm expressed using arrays, potentially due to some non protein-coding genes that were present on arrays, and/or genes that are obsolete and no longer part of the most recent Araport11 annotation.

### Isoform level quantification in Arabidopsis gametes

Transcriptome analysis using RNA-seq facilitates expression quantification not only at the gene level but also at the isoform level. To quantify isoforms, we first performed a de novo transcriptome assembly using SC and VN data produced in this study, in combination with published datasets from 10 other tissues, focusing mostly on reproductive tissues (Supplemental Table 2). We did this because we anticipated that the Arabidopsis gametes, both male and female, may potentially express a set of specific transcripts not previously identified in any other tissues studied. In addition, the most recent Arabidopsis Annotation, Araport 11 (Cheng et al., 2017), was based on vegetative tissues and various stress treatment RNA-seq data, and lacks data, particularly from reproductive cell types such as sperm, ECs, and VN, as those were unavailable at the time. This suggests some novel isoforms might be missing even in the recent most comprehensive Araport annotation. Thus, we used a total of 1.17 billion reads to reassemble the Arabidopsis transcriptome (Supplemental Table 2).

The transcripts were assembled for each sample using Stringtie. Across all 12 tissue/cell types a total of 58,039 nonredundant transcripts were generated, mapping to 27,655 loci, and with 20,861 (75%) gene models containing at least one intron. We recovered a total of 48,358 known transcripts and 9,681 novel transcripts. For this study, only protein-coding genes were considered for AS analysis.

If we focus on the three cell types in this study, the number of expressed transcripts (average TPM ≥ 1) ranged from 11,113 (7,135 loci) in SCs, over 15,265 (8,916 loci) in VN to 25,222 (14,805 loci) transcripts in ECs. This annotation was then used to quantify the isoforms detected. We found that 42% of multi-exonic genes undergo AS in SCs and VN. This number was slightly higher in ECs (53%) (Supplemental Table 3). Overall, 12,658 alternatively spliced genes gave rise to 25,023 AS events in our assembled transcriptome. Out of these isoforms predicted, 2,091 were expressed in SCs, 3,600 in VN, and 4,530 in ECs. To identify how many of these transcripts were specific to each cell type, we calculated a specificity score called specificity measure (SPM). This ranges between 0 and 1 and the larger the SPM values, the higher the tissue specificity. Using the stringent specificity score of SPM ≥ 0.9 (Carmargo et al., 2020), we found 1,174 (386 novel), 1,928 (736 novel), and 1,813 (568 novel) transcripts to be specific to SC, VN, and EC, respectively. Sequence analysis of overrepresented splice sites (SSs) at alternative 5´ SS and 3´ SS in the assembled transcriptome revealed that these SSs were associated with GU and AG dinucleotides (Supplemental Figure 1).

The relatively high number of germ cell-specific novel transcripts identified here indicates that the Arabidopsis annotation remains incomplete. It is heavily biased toward vegetative tissues, and more condition and/or cell/tissue-specific transcripts could be missing.

### Cell-type-specific alternatively spliced genes

**Figure 3:**
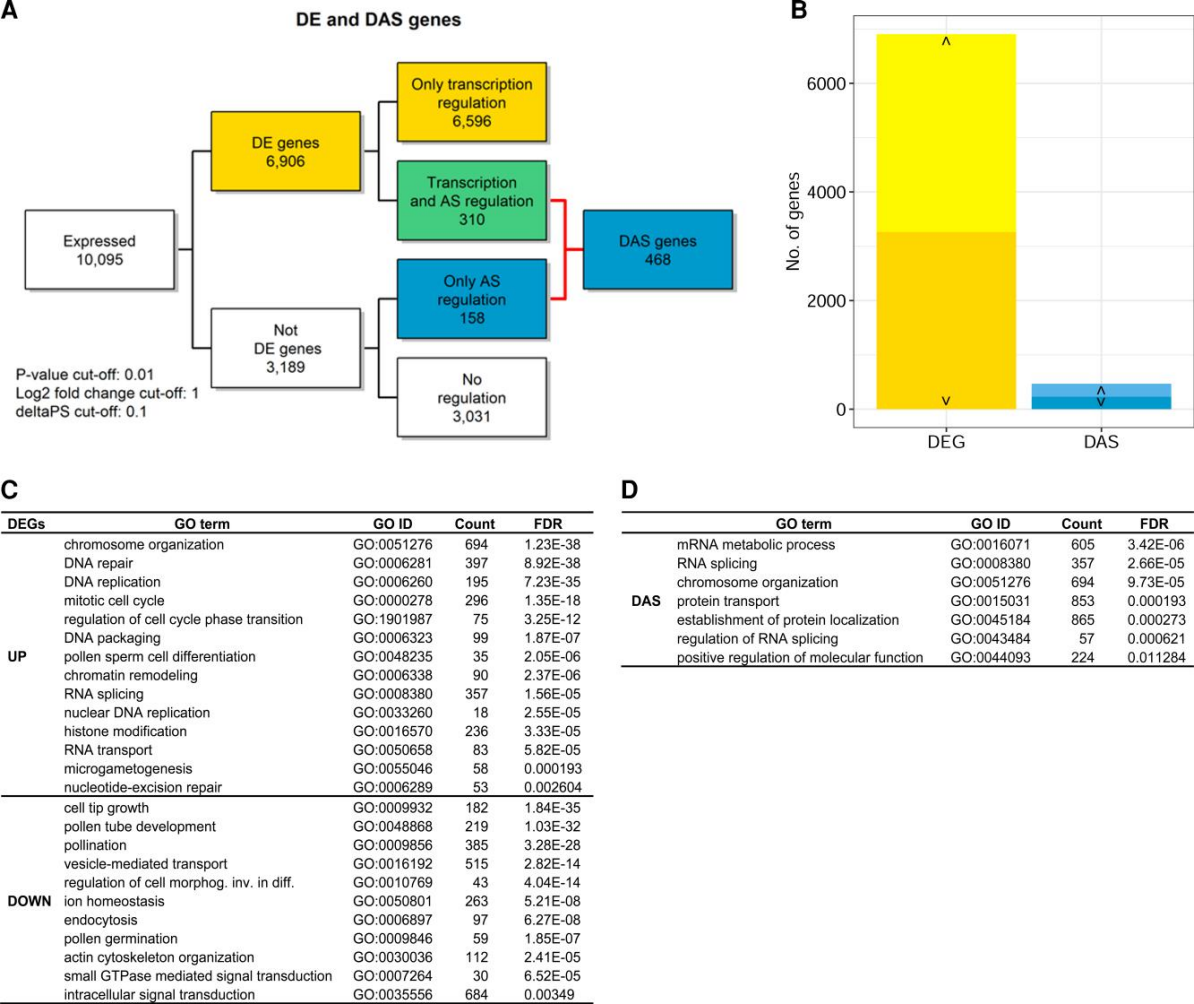
Differential expression analysis at gene and transcript level. A, Flowchart depicting analysis of gene expression and AS between SCs and VN, including numbers of DE and DAS genes. B, Bar plots showing the number of DE and DAS genes, separated by upregulated (∧) and downregulated (∨). C, Functional enrichment of DE genes between SC and VN. D, Functional enrichment of DAS genes between SC and VN. For details, see Supplemental Table 5.

To determine alternatively spliced genes that were differentially regulated between male and female gametes, we used limma in the 3D RNA-seq pipeline (Guo et al., 2021) to identify the differentially expressed (DE) and differentially alternatively spliced (DAS) genes using pairwise comparisons: between SC and VN, and between SC and EC, both at gene level as well as transcript level. When the transcriptome of SC was compared with the VN, we identified 6,906 DE genes (FDR < 0.01 and log2 fold change > 1), and 468 DAS genes (FDR < 0.01 and percent spliced (ΔPS) > 0.1) (Figure 3, A and B) (Supplemental Table 4). When we compared the genes that were either differentially expressed or spliced between SC and VN, a substantial overlap of 310 genes was observed (Figure 3A).

These genes might be regulated both at transcriptional as well as splicing levels. The 468 DAS genes encompassed 2,131 transcripts, out of which 1,411 (66.2%) were known transcripts, while 720 (33.8%) comprised previously unidentified transcripts. Similarly, when we compared SC and EC, we identified 10,809 DE genes and 608 DAS genes (Supplemental Table 4), with 416 genes showing regulation both at transcription and splicing levels (Supplemental Figure 2, A and B).

### Functional classification of DE genes and transcripts

Next, we asked ourselves what could be the function of these DE genes. A gene ontology analysis of the 6,906 DE genes in our sperm versus VN comparison, indicated the GO categories cell-cycle regulation, DNA metabolic process, and chromatin organization as having the highest enrichment scores for the upregulated genes in SCs (Figure 3C) (Supplemental Table 5). The genes that were downregulated in SCs were associated with pollen germination and tube growth, and vesicle-mediated transport. On the other hand, gene set enrichment analysis of DAS genes between sperm and VN revealed mRNA splicing, chromatin modification, and protein localization and transport as significantly enriched terms (Figure 3D).

We then tried to understand the differences between SCs and ECs, and we found 10,809 genes to be differentially expressed between the two cell types. Our functional enrichment analysis of 3,804 genes that were upregulated in SCs revealed chromosome organization, DNA replication, and cell cycle and chromatin organization as significant terms (Supplemental Figure 2C), similar to the results obtained for the SC versus VN upregulated genes. Genes that were differentially spliced between sperm and EC were found to be functionally enriched for mRNA processing, RNA splicing, and regulation and metabolic process (Supplemental Figure 2D).

### SCs and VN differ in their splicing landscape

**Figure 4:**
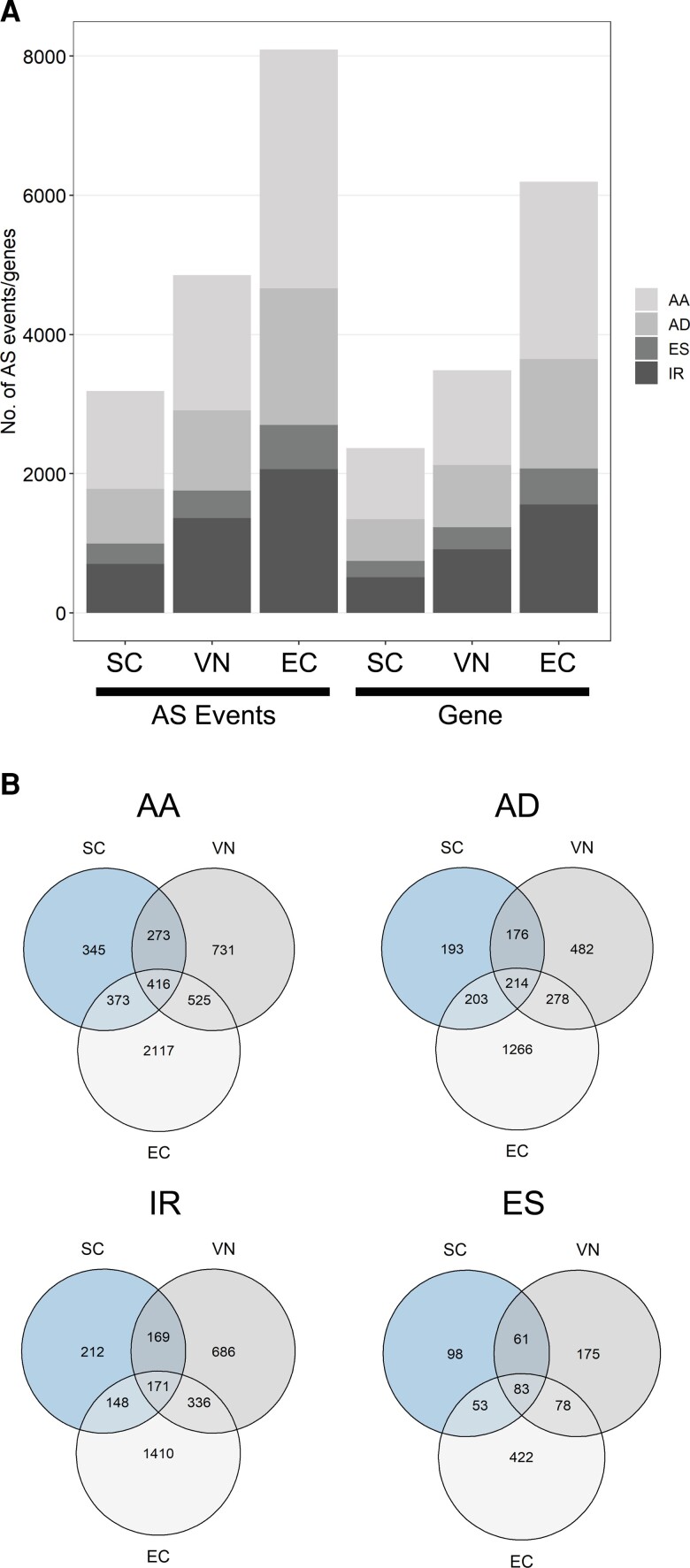
Sex-specific AS in the Arabidopsis germline. A, Bar plot showing the distribution of four predominant forms of AS events in SC, VN, and EC, respectively: ES, exon skipping; IR, intron retention; AA, alternative acceptor site; AD, alternative donor site. The four bars on the right depict the corresponding number of genes undergoing these four different types of AS. B, Venn diagrams depicting the number of overlapping AS events between the three cell types. For details, see Supplemental Table 6.

When comparing AS studies in plants and animals, it is evident that information about tissue-specific AS is relatively sparse in plants. Moreover, the relative proportion and frequency of different types of AS events in plants and animals differ (Chaudhary et al., 2019). To analyze the AS landscape in germ cells and determine their cell-specific pattern in SCs, VN, and ECs, we used the Suppa2 tool (Trincado et al., 2018) and classified the splicing events detected into four types: intron retention (IR), exon skipping (ES), alternative acceptor (AA), and alternative donor (AD) (Figure 4).

The alternative acceptor type was found to be the most predominant AS event in all three cell types, followed by intron retention in the VN and EC, whereas in SCs, alternative donor was the second most prevalent form of splicing events (Figure 4A). The least abundant splicing event in all three cell types was found to be exon skipping. We also looked at the common AS events shared between the three cell types and identified 884 common AS events corresponding to 634 genes. These common AS events consisted of 171 IR events, 416 AA events, 214 AD events, and 83 ES events (Figure 4B) (Supplemental Table 6).

Tissue-specific AS data are limited in plants, more so in other reference plant species, such as rice and maize. To understand whether the AS landscape in germ cells is different between monocots and dicots, we used published data sets of sperm, VN, and ECs from rice and maize as a reference for monocot species (Anderson et al., 2013; Chen et al., 2017) and reanalyzed these to quantify the splicing landscape in other species.

**Figure 5:**
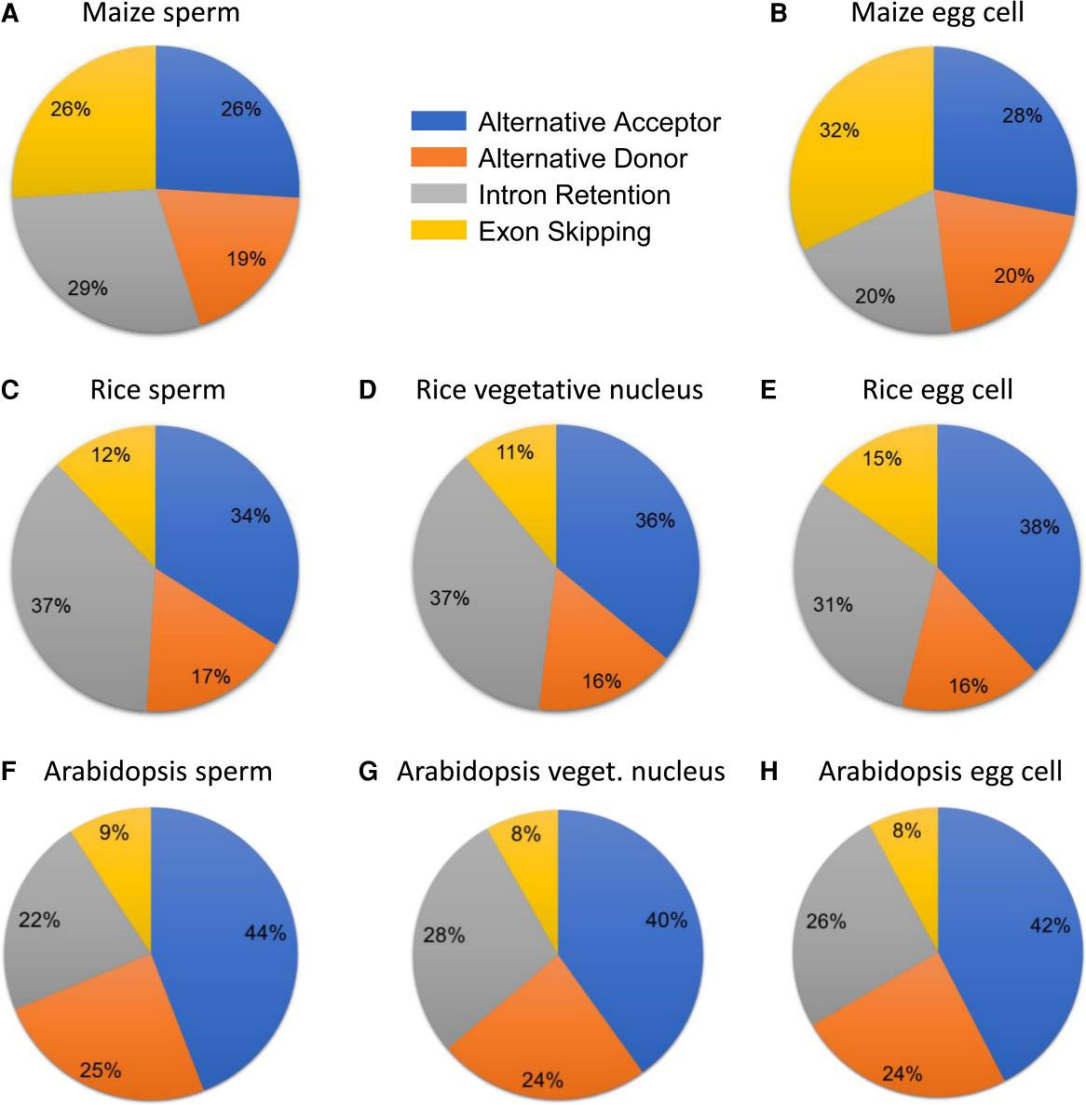
Comparison of AS landscape in germ cells of several plant species. A,B, AS landscape in maize SC and EC, respectively. C–E, AS landscape in rice SC, VN, and EC. F–H, AS landscape in Arabidopsis SC, VN, and EC.

Surprisingly, our analysis revealed that the proportion of AS events in gametes of rice and maize is different from that observed in Arabidopsis (Figure 5). We found exon skipping to be the most predominant form of splicing event in maize ECs and the second most prevalent in SCs (Figure 5, A and B), in contrast to both rice and Arabidopsis. Intron retention is the most predominant form of splicing event in the rice male germline (Figure 5, C and D), however alternative acceptor is the most predominant form of splicing event in ECs of rice (Figure 5E), similar to what we observe in Arabidopsis. Taken together our data of these three species indicate that AS is highly tissue and condition-specific, and probably more complex in plants than previously thought.

### DAS genes show variable expression between SC and VN

**Figure 6:**
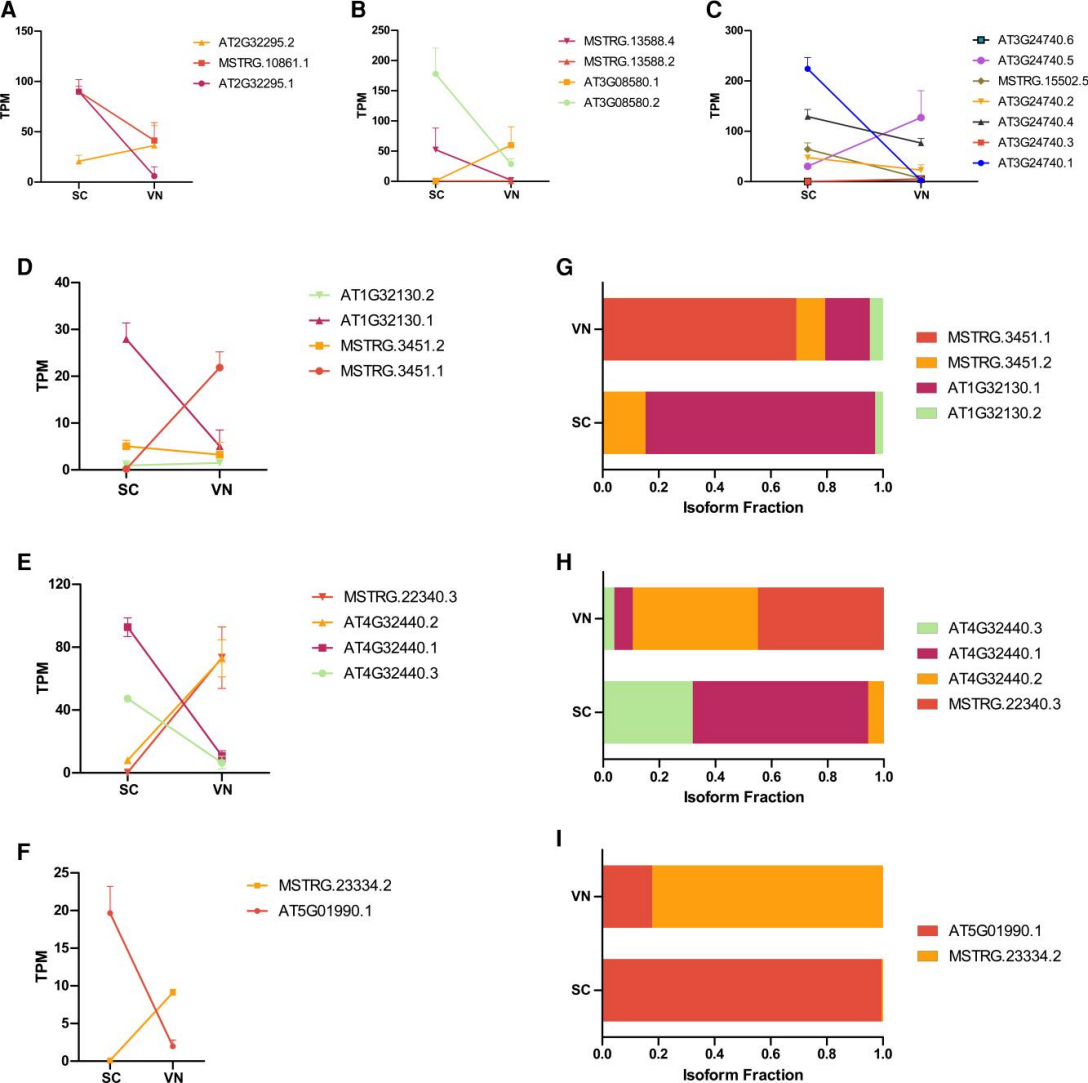
Isoform level dynamics in SCs and VN. A–C, Expression level (TPM) of individual isoforms that are differentially expressed between SCs and VN, representing examples of complex splicing patterns. D–F, Expression levels (TPM) of isoforms of genes depicting simple cases of isoform switching. G–I, Changes in the isoform fraction of the corresponding genes are shown in (D–F). The error bar indicates the Sd across three biological replicates (*n* = 3).

Our detailed comparison of the DAS genes between SCs and VN at the isoform level revealed varying expression dynamics ranging from complex splicing patterns (Figure 6, A–C) to simple isoform switching (Figure 6, D–F). Although a complex splicing pattern was observed for many genes, we could only detect 26 instances of isoform switching when we performed pairwise comparisons between SC and EC, and SC and VN (Supplemental Table 7).

Instances of simple isoform switching often showed very small changes in total gene expression despite substantial differences in the abundances of individual isoforms. For instance, if we look at the expression of AT1G32130 at the gene level, with two annotated (AT1G32130.1 and AT1G32130.2) and two novel transcripts (MSTRG.3451.1 and MSTRG.3451.2), no substantial difference was observed between sperm and VN (Figure 6D). However, measurement of isoform level expression revealed that the canonical isoform (AT1G32130.1) was more expressed in SCs and downregulated 5.5-fold in VN, whereas the novel transcripts MSTRG.3451.1 and MSTRG.3451.2 were downregulated by more than 100-fold in SCs, thereby showing completely opposite expression level in SCs and VN. On the other hand, if we look at the gene expression level change of AT5G01990, with one annotated and one predicted isoform, we could see the differences both at the gene expression level (downregulated by 1.78-fold in the VN) as well as at the isoform level (Figure 6F). Complex splicing often resulted in a dynamic expression of isoforms, which in turn resulted in a net shift in the expression of the gene toward the direction of the predominant isoform (Figure 6, A–C).

**Figure 7:**
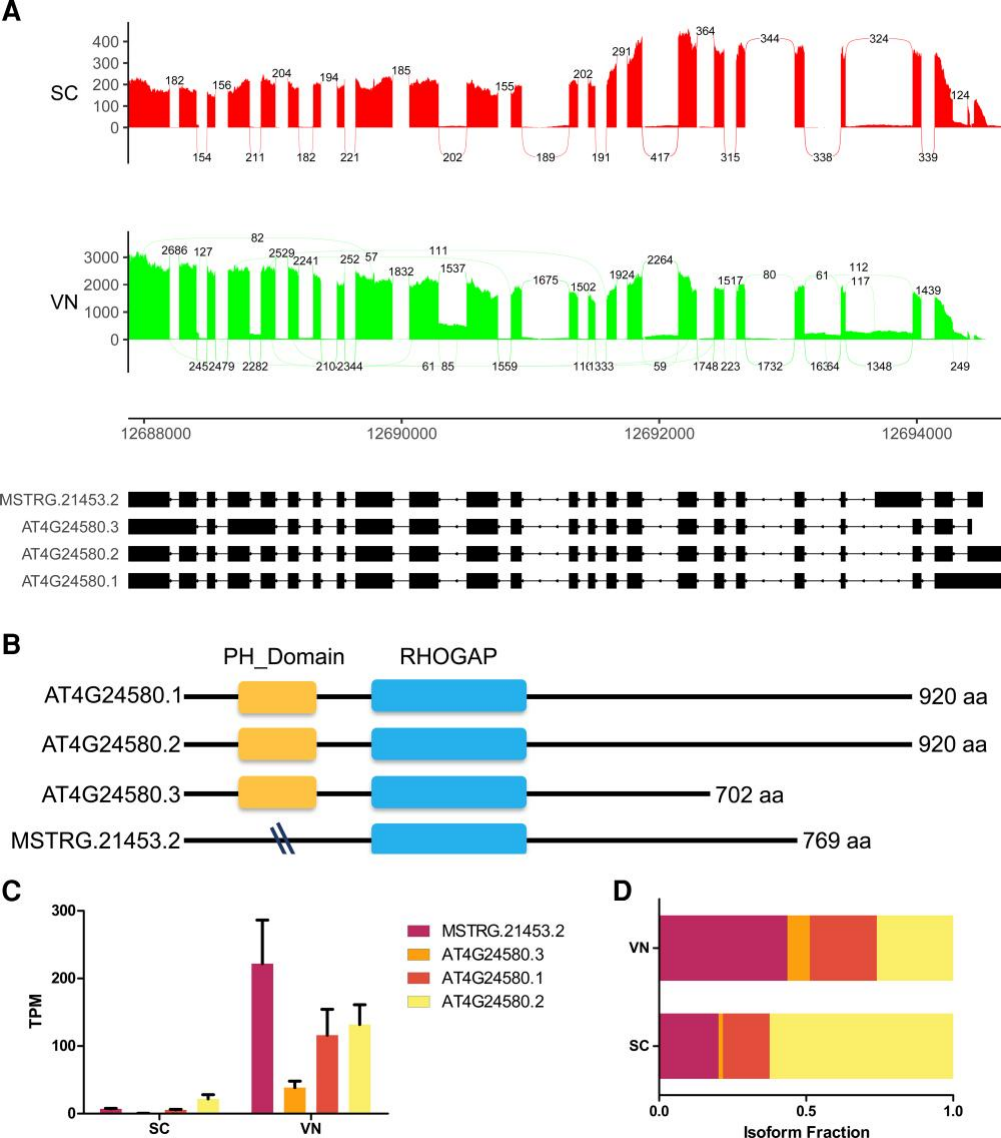
AS of the Rho GTPase-activating gene REN1 (AT4G24580). A, Sashimi plot showing AT4G24580 gene generating four different transcript isoforms detected with our RNA-seq pipeline. Peaks in red (SC) and green (VN) represent read coverage, and curved lines with numbers in red and green represent splice junctions supported by that number of reads (*Y*-axis). For each transcript isoform, the blocks in black represent exons, and the lines between the blocks represent introns. The number at the base of the sashimi plot represents the genomic coordinate. B, Protein length and domain predictions for the respective isoforms. C, Expression level (TPM) of individual isoforms that are differentially expressed and spliced between SCs and VN. D, Changes in the isoform fraction of the corresponding gene shown in (C). The error bar indicates the Sd across three biological replicates (*n* = 3).

Many of these predicted transcripts are in fact expressed at a much higher level compared to the canonical transcripts in our analysis (Figure 7, C and D). For instance, our analysis of splicing in SCs and VN revealed that the gene *REN1* (*ROP1 ENHANCER 1*) has three annotated transcripts and one novel predicted transcript (MSTRG.21453.2), with the latter showing higher expression in the VN than the annotated ones (Figure 7A). *REN1* encodes a Rho GTPase-activating protein (RhoGAP) and is required for normal pollen tube growth (Hwang et al., 2008). The gene was found to be both differentially expressed and spliced between sperm and VN. Our analysis also revealed that a novel isoform MSTRG.21453.2 had an AS pattern and encoded for a protein smaller than the canonical isoforms (Figure 7B). This isoform lacks the PH domain, indicating that splicing may have a profound impact on the proteome as well. Moreover, the predicted isoforms can be further analyzed to characterize specific splicing events and potential consequences. Many of these different splicing patterns can result in potential consequences both at the RNA and protein level (Figure 7). One of the potential consequences is isoforms with premature termination codons that might result in nonsense mediated decay (NMD) (Supplemental Figure 3–5). This is one of the several ways, how gene expression is regulated (Rayson et al., 2012; Nickless et al., 2017). Nevertheless, more functional studies are needed to understand the biological importance of the loss of domains in these kinds of protein isoforms as well as role of the NMD in gene expression regulation.

### Sex-specific regulation of transcription and splicing factors

The Arabidopsis male gametophyte undergoes extensive transcriptome reprogramming during pollen development and studies so far suggest that this is a tightly regulated process with at least two major successive gene expression reprogramming events occurring during pollen development (reviewed in Rutley and Twell, 2015). However, to which extent AS shapes this reprogramming during the two successive pollen mitoses remains unknown. Moreover, knowledge about transcriptional control at the specific level of SCs and VN remains limited. To bridge this gap, we used our RNA-seq data to identify various TF families expressed in the SCs and/or VN and compared them with expression in the female germline.

**Figure 8:**
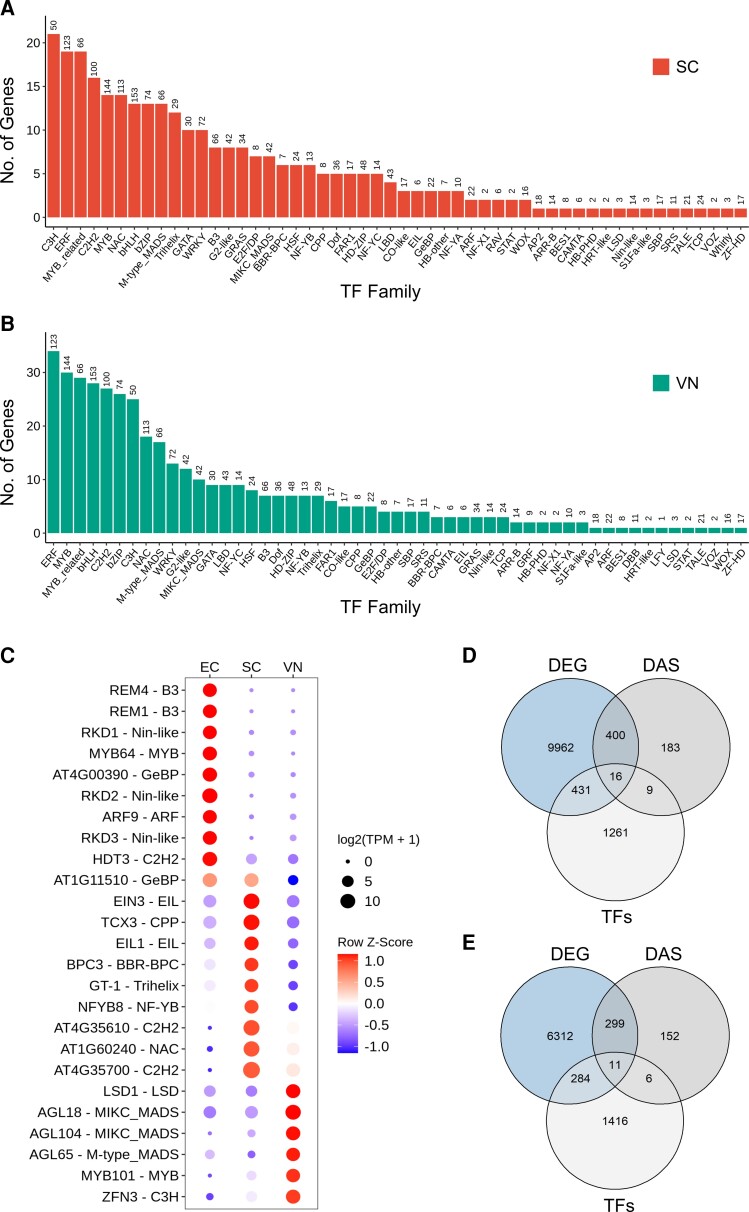
Transcriptional regulation in male and female germlines. Number of TFs per family expressed in SC (A) and VN (B). The number on top of each bar indicates the total number TFs genes present in a given family. C, Dot plot showing the expression of 25 most variable TFs across the three cell types. D, Venn diagram showing the number of TFs differentially expressed (DEG) or spliced (DAS) between SC and EC. E, Venn diagram showing the number of TFs differentially expressed or spliced between and SC and VN. For detailed information, see Supplemental Table 8.

Using the plant TF database (http://planttfdb.gao-lab.org/; Jin et al., 2017), we analyzed the expression of a total of 1,717 annotated TFs, belonging to 53 different TF families. The most predominant TF families in SCs were C3H (*n* = 21) and ERF (*n* = 18), whereas in VN the most predominant TF families were ERF (*n* = 33), MYB (*n* = 29), and MYB-related (*n* = 28) (Figure 8, A and B). On the other hand, the most predominant TF family in ECs were bHLH (*n* = 41) and ERF (*n* = 41) (Supplemental Figure 6). Our results also indicate that the expression of several TFs was specific to each cell type (Figure 8C).

Out of 1,717 annotated TFs, 295 TFs belonging to several different TF families were differentially expressed when we compared SC and VN. At least 17 TFs were also regulated at the splicing level, of which 6 exclusively at the splicing level, suggesting that regulation of the male germline in Arabidopsis occurs not only at the transcriptional level, but also to a certain extent at the splicing level (Figure 8E). We made a similar observation when comparing SC and EC. Here, 437 TFs were differentially expressed, whereas 25 TFs were regulated at the splicing level, and 9 of those exclusively at the splicing level (Figure 8D) (Supplemental Table 8). Overall, our analysis sheds light on cell-type-specific transcriptional control of both male and female germlines, with several TFs being exclusively expressed in specific cell types, marking them as potential candidates for further functional analyses.

**Figure 9:**
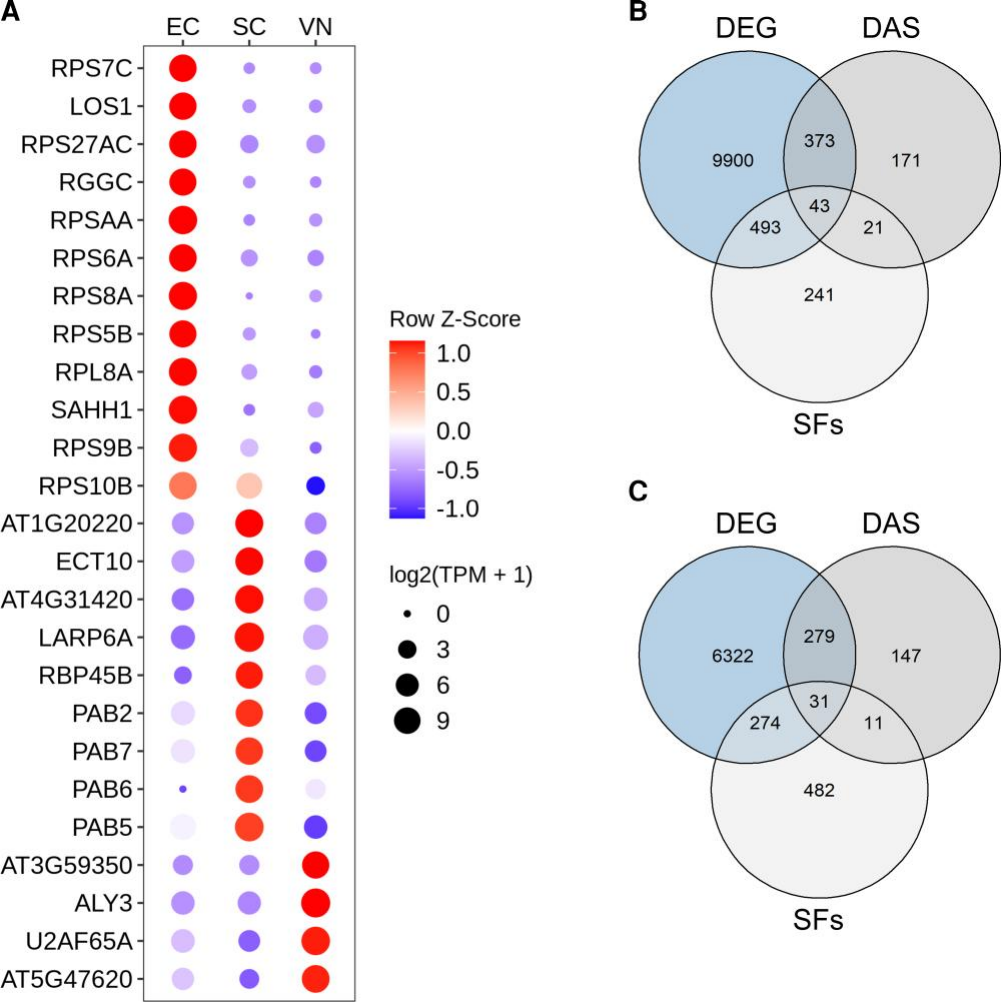
Splicing regulation in the three cell types. A, Dot plot showing the expression of 25 most variable splicing factors across the three cell types. B, Venn diagram showing the number of splicing factors differentially expressed (DEG) or spliced (DAS) between SC and EC. C, Venn diagram showing the number of splicing factors differentially expressed or spliced between SC and VN. For detailed information, see Supplemental Table 8.

AS mechanisms involve spliceosome proteins and splicing factors, which in addition might undergo AS themselves. The Arabidopsis genome encodes for multiple splicing factors/RNA-binding proteins, and our results show that many of these genes are differentially expressed or spliced in a gamete-dependent manner. In our SC versus VN comparison, we found that at least 274 splicing factors were differentially expressed, and 42 were differentially spliced (Figure 9C) (Supplemental Table 8). Similarly, when we looked at SC and EC, 536 splicing factor genes were differentially expressed, and 64 were differentially spliced between SCs and ECs (Figure 9B) (Supplemental Table 8). Interestingly, 11 and 21 splicing factors were only regulated at the splicing level when we compared sperm and VN or SC and EC, respectively (Figure 9, B and C).

Similar to what we observed for TFs, the splicing machinery seems to be unique to each cell type and several splicing factors were found to be exclusively expressed in one of the cell types (Figure 9A), potentially mediating sex-specific functions during plant reproduction. Splicing factors are known to undergo splicing themselves to mediate their response in a tissue-dependent manner, but so far no study has reported their role in plant germline development and our data could therefore provide a good starting point for such future studies.

## Discussion

Male gametophyte development in flowering plants has been a very active area of research over the last two decades. Extensive transcriptomic and epigenomic data sets have provided unprecedented insights into pollen development (Borges et al., 2008; Slotkin et al., 2009; Calarco et al., 2012; Ibarra et al., 2012). The wealth of data available is not just limited to Arabidopsis, but rice, maize, and more recently tomato have also been studied (Anderson et al., 2013; Chen et al., 2017; Liu et al., 2018). The transcriptomic data available for pollen development vary though between species. For instance, in Arabidopsis data from every stage of pollen development from unicellular microspore to mature pollen grain are available (Honys and Twell, 2004; Julca et al., 2021), while for most other species the data are largely limited to mature pollen grains. Similarly, when it comes to SCs, data from Arabidopsis, rice, maize, and tomato are available. However, VN data are only available for rice (Anderson et al., 2013). This might be because the isolation of VN from pollen grains is technically challenging. Also, it is important to note that, while the Arabidopsis SC transcriptome was based on microarrays, rice, tomato, and maize, germline transcriptomes are available in the form of RNA-seq data as well. Only recently, RNA-seq data on Arabidopsis SCs were published, but without detailed analysis (Borg et al., 2020). We closed this gap by performing RNA-seq analysis on SCs and VN isolated from mature pollen grains of Arabidopsis. This allowed us not only to identify genes expressed in SCs and VN on a true whole-genome level but also to uncover genome-wide AS patterns in the germline. The transcriptome data from SCs helped to identify 21% more genes as expressed than previously known (Figure 2D). Our transcriptomic data from VN also revealed a higher number of genes than previously reported for whole pollen grains. Thus, overall our study revealed the transcriptome of the individual components of the mature pollen grain at a much higher resolution than can be achieved when isolating RNA from whole pollen grains.

Transcriptome reprogramming during pollen development has been well studied but most of the studies have focused on changes at the gene expression level. However, with RNA-seq it is possible to identify changes not only at the gene level but also at the transcript level, revealing the scale of AS in reproductive cell types. The first landmark transcriptomic study to identify changes in pollen at the splicing level was published by Loraine et al., (2013), in which 30 genes were found to be differentially spliced between leaf and pollen. However, this study could not reveal the relative contribution of transcripts coming from SC or VN. Our study, therefore, served to fill this knowledge gap toward the understanding of genome-wide AS in germ cells. To guide our analysis, we used a combination of de novo assembly and reference mapping to reassemble the Arabidopsis transcriptome, combining our datasets with published RNA-seq data, particularly from reproductive stages. We identified 58,039 transcripts, an increase of approx. 20% over the number of transcripts predicted in Araport 11, the most recent Arabidopsis annotation comprising 48,358 protein-coding transcripts. In addition, when we compared the Araport 11 annotation with our assembled transcriptome, we identified at least 2,881 genes that were previously not known to undergo splicing. Compared to the more recent, updated Arabidopsis annotation AtRTD2 with its 74,194 nonredundant protein-coding transcripts (Zhang et al., 2017), we were still able to identify 2,148 genes that were not predicted to undergo splicing. This is in line with the recent identification of 1,601 new transcripts by Raxwal et al., (2020) and suggests that the Arabidopsis annotation is still far from complete and more tissue and condition-specific transcripts might await discovery. Furthermore, more recently the updated version of AtRTD3 was published, which is by far the most comprehensive Arabidopsis Reference Transcript Dataset containing over 169,000 transcripts (Zhang et al., 2022). We compared our annotation to AtRTD3 and were still able to find ∼550 genes that were undergoing AS exclusively in our dataset (Supplemental Figure 7D). Interestingly, many of these over 550 genes were associated with functions associated with plant reproduction, such as pollen development and SC differentiation, further indicating the utility of our dataset covering more specialized cell types such as SCs, VN, and ECs. Being aware that predictions of new splicing forms are not completely error-free, we applied the most stringent criteria at every step of AS analysis to minimize the detection of false positive or false negative transcripts and AS events. Also, to further validate, we compared the annotation from this study to Araport 11 and AtRTD3 based on long read sequencing and found that predicted gene models from our study match well with both AtRTD3 and Araport11 (Supplemental Figure 7).

To provide robustness to our dataset, we have experimentally validated some of the predicted transcripts found in our analysis (Supplemental Figures 8–12). The fact that in some cases additional alternative transcripts were observed by RT-PCR shows that applying too stringent criteria can lead to loss of detection sensitivity. The future use of more advanced RNA-seq techniques, such as long-read sequencing (ISO-seq), could help to identify additional biologically relevant isoforms in a specialized cell type, such as SCs. Currently, these long-read sequencing protocols still require relatively large quantities of input, which is technically challenging to obtain from small specialized cell types such as SCs, VN, or ECs.

The role of splicing factors and RNA-binding proteins in plant reproduction is relatively unexplored. Splicing factors are commonly known to be involved in stress responses. However, recently some of them have been reported to have a role in reproduction. For instance, SR45 and U2AF65A/B have been implicated in pollen germination and tube growth (Cao et al., 2013; Park et al., 2019). Arabidopsis has 798 known splicing factors/RNA-binding proteins (Calixto et al., 2018). This complex network of splicing factors and RNA-binding proteins might have an important function in plant reproduction and could be an area worth exploring for future research. Our transcriptome data from SCs also helped to identify the expression of 76 additional splicing factors for which there were no probes on microarrays (Borges et al., 2008).

Information about tissue-specific splicing in Arabidopsis is very limited and the only study on Arabidopsis pollen provided no information about the different types of splicing events. We showed that the most predominant form of AS event was alternative acceptor in all three cell types, followed by intron retention in VN and EC, while in SCs alternative donor was the second most predominant form of splicing (Figure 4A). However, a recent study on AS in 28 different tissues (flowers, shoots, seeds, roots, and leaves) and developmental stages in soybean (Shen et al., 2014) points toward differences in the splicing landscape between monocots and dicots. To test this hypothesis, we compared AS in SCs and VN of Arabidopsis with that in two reference monocot species, rice, and maize. Our data revealed that the relative proportion of AS events between monocot and dicot shows a high degree of variation, particularly in maize, where exon skipping was found to be the most prevalent (32%) and second most (26%) prevalent form of AS event in EC and SC, respectively (Figure 5, A and B). This difference in the proportion of splicing events between maize and the other two species (Arabidopsis and rice) might be species-specific. Recently, developing ears in maize were shown to undergo exon skipping as their most predominant form (27%) of splicing event (Thatcher et al., 2016), a form usually expected to be the least abundant in plants. We found intron retention to be the most predominant form of splicing in rice sperm and VN, which is in contrast to our results in Arabidopsis. Interestingly, we did not find intron retention to be the most predominant form of AS events as is generally expected in plants, particularly in Arabidopsis. Intron retention is thought to be overestimated in plants, a notion that has recently been validated in other studies (Martín et al., 2021) as well as in our analysis. These differences could be explained at least partly, due to the advances in RNA-seq technologies and improvement of de novo assembly algorithms and genome annotation detail that may help to improve the prediction of transcripts and splicing events. Nevertheless, how the expression dynamics and splicing patterns of different splicing factors contribute to the overall fertility of plants is unclear and calls for further research.

Our analysis of sperm and VN transcriptome also revealed more than 6,000 genes to be differentially expressed. Genes that were upregulated in SCs were enriched for DNA replication and packaging, SC differentiation, and cell-cycle progression (Figure 3C). The functional enrichment analysis points toward the role of genes that are involved in SC maturation before gamete fusion and is similar to previous classifications of SC-enriched transcripts (Borges et al., 2008). Sperm transcriptome analysis in other plant species like rice, maize, and tomato has also revealed enrichment of transcripts with similar functions, such as cell-cycle regulation, proteasome-mediated protein metabolism, and DNA repair and replication (Anderson et al., 2013; Chen et al., 2017; Liu et al., 2018). This indicates that the process of sperm maturation and differentiation is largely conserved across flowering plants. Genes that are downregulated in SCs relative to the VN are involved in pollen germination and tube growth, cell–cell communication, and vesicle-mediated transport. These functions are important for pollen tube growth and hence reflect the main function of the VN.

Pollen development is under tight transcriptional control and several TFs have been identified to be important for pollen development, germination, and ovule targeting (Rutley and Twell, 2015). Our analysis identified at least 295 TFs that were differentially expressed between SCs and the VN (Figure 8E). TFs are not only regulated at the gene expression level, but also at the splicing level, and at least six are exclusively regulated at the splicing level, warranting further functional characterization. Several TFs were found to have cell-type-specific expression patterns (Figure 8C), some of which have already been studied. For instance, agamous-like genes *AGL65, AGL66* and *AGL104* belonging to the MIKC subgroup of the MADS-box TF family were found to be preferentially expressed in mature pollen grains (Pina et al., 2005). Functional characterization of *agl65*, *agl104*, *agl66*, and *agl94* in different combinations of double and triple mutants highlighted the role of MADS-box TF networks for pollen development and maturation (Verelst et al., 2007; Adamczyk and Fernandez, 2009).

Extensive studies of male germline development over the past years have highlighted the role of the transcriptional regulon mediated by the MYB TF DUO1. Our analysis of cell-specific TFs points toward a DAZ3 (DUO1-ACTIVATED ZINC FINGER 3) C2H2 type TF to be highly specific to SCs, and it is predicted to be a direct target of DUO1 (Borg et al., 2011). Similarly, the RKD subfamily in Arabidopsis has five genes, out of which three were found to be highly expressed in ECs. Interestingly, although several T-DNA insertion alleles of Arabidopsis *RKD* genes are available, no EC defects have been reported so far for *rkd1/2* double mutants, suggesting high levels of functional redundancy among gene family members or lack of effective functional alleles. The use of gene editing techniques such as CRISPR and deleting all genes to uncover a potential EC defect may help to provide more detailed insights into this predominantly EC expressed subfamily (Wuest et al., 2010; Kőszegi et al., 2011).

In conclusion, using RNA-seq, we have shown the extent of genome-wide AS occurring in the SC and VN. In addition, our comparative analysis of the male and female germline helped to shed light on sex-specific modulation of AS, an area not addressed in previous studies. Our annotation helped to predict at least 20% more transcripts than previously known, including many transcripts that are highly specific to SC, VN, or EC. Many of these genes are also associated with transcription, splicing and chromatin modification, and are differentially spliced between SC and EC. These new splice variants potentially contribute to proteome diversity and function in Arabidopsis gametes. Our study, therefore, provides an overview of genome-wide AS in SC, VN, and EC and serves as a starting point for further in-depth analysis at functional and molecular levels.

## Materials and methods

### Plant material and growth conditions

Seeds of a transgenic Arabidopsis (*Arabidopsis thaliana*) marker line harboring MGH3p::MGH3-GFP and ACT11p::H2B-mRFP (Borges et al., 2012) were sown on soil in short-day conditions (8-h light at 21–23°C) for 8 weeks and then transferred to long-day conditions (16-h light) to induce flowering.

### RNA-seq library preparation and reference-based transcriptome reassembly

Fluorescence activated cell sorting was used to sort SC and VN from mature pollen (Borges et al., 2012; Santos et al., 2017). Five hundred cells/nuclei were sorted each for SC and VN and for each replicate respectively, in lysis buffer. Sorting was performed in batches, with 500 cells/nuclei in one single tube, and full-length Smart-seq2 cDNA libraries were produced as described in (Misra et al., 2019).

cDNA libraries were checked on a Fragment Analyser for quality and yield and then converted into Nextera libraries at the Genomics Facility of Instituto Gulbenkian de Ciência, Oeiras, Portugal, and sequenced at Novogene, United Kingdom, with 150 bp paired-end reads on an Illumina Novaseq. The reads were checked for quality and then mapped to the Arabidopsis genome (TAIR 10) assembly using Hisat2 v2.1.0 with command line options “–dta –min-intronlen 60 –max-intronlen 6000” (Kim et al., 2019).

Transcriptomes from each RNA-seq library were reconstructed using reference-based transcriptome assembly by Stringtie v2.0 using the parameters -c 5 -a 15 -j 10 (Pertea et al., 2015). The assembled transcripts were merged to form a master annotation using the merge option of Stringtie. The merged annotation was compared to reference annotation using gffcompare and the assembled transcripts were then identified as known or novel isoforms based on class code annotation (Pertea et al., 2016). In the most recent annotation, while the known transcripts retained the names of genes from the reference Araport11 annotation, the novel transcripts were named according to Stringtie's default naming convention (e.g. MSTRG.1090.1). Only transcripts having an expression of TPM ≥ 1 were retained for downstream analysis.

### Identification of differential gene expression and splicing

Differential expression and AS analyses were carried out using 3D RNA-seq App (Guo et al., 2021). The transcriptome assembly was used to quantify TPM using Salmon (version 0.14.1) for each sample (Patro et al., 2017). Low-expressed genes or transcripts were filtered out, and only those genes were retained that had an expression of 1 cpm (count per million ≥1) in at least two samples. Normalization of expressed transcripts was performed using the trimmed mean of *M*-values method, followed by pairwise analyses.

Differential gene expression analysis between two groups was performed using limma pipeline with thresholds of adjusted *P* < .01 and fold change log2FC >1. To identify the DAS genes between two contrast groups, adjusted *P* <.01 and at least one transcript of the gene with percent spliced-in ΔPS > 0.1 were used. AS sashimi plots across a gene were generated from RNA-seq data using ggsashimi (Garrido-Martin et al., 2018).

### Gene ontology analysis

Functional enrichment of genes that were differentially expressed or spliced in pairwise comparisons was determined using an updated version of g:Profiler (Raudvere et al., 2019), with thresholds of adjusted *P*-value <.001 and <.05, respectively (significance threshold method: g_SCS).

### Detection of AS events

Different types of AS events were identified using Suppa2 (Trincado et al., 2018). Major AS events were classified into intron retention (IR), exon skipping (ES), alternative 3' SS or alternative acceptor (A3SS/AA), and alternative 5′ SS or alternative donor (A5SS/AD). To identify isoform switching events, we used default parameters of the 3D RNA-seq App that uses the salmon output to detect significant isoform switches between conditions using the inbuilt IsokTSP tool (Sebestyén et al., 2015; Guo et al., 2021).

### RT-PCR validation

Independent samples of 500 FACS sorted SCs and VN, respectively, were used for RT-PCR validation. The sorted cells were subjected to the Smart-seq2 protocol as described above. cDNA libraries obtained from SCs and VN were then used as templates for PCR using gene-specific primers (Supplemental Table 9) with the following PCR conditions: 2 min at 95°C, followed by 37 cycles of 30 s at 95°C, 45 s at 56°C, and 1 min at 72°C, followed by 5 min at 72°C.

## Accession numbers

All fastq raw RNA-seq files for SCs and VN samples are deposited in the NCBI GEO database under accession number GSE201115.

## Supplemental data

The following materials are available in the online version of this article.

**Supplemental Figure S1**. Sequence logos of the exonic and the intronic boundaries of the 5´ and 3´ SSs.

**Supplemental Figure S2**. Differential expression analysis at gene and transcript level between sperm cell and egg cell.

**Supplemental Figure S3**. Overview of the isoforms predicted to have functional consequences in AT2G25910.

**Supplemental Figure S4**. Overview of the isoforms predicted to have functional consequences in AT4G00660.

**Supplemental Figure S5**. Overview of the isoforms predicted to have functional consequences in AT2G26540.

**Supplemental Figure S6**. Number of transcription factors (TFs) per family expressed in egg cell (EC).

**Supplemental Figure S7**. Visualization of predicted gene models on IGB for comparison between Araport11, AtRTD3 and this study for four representative genes: AT1G61140, AT1G75560, AT3G01330, and AT3G22360.

**Supplemental Figure S8**. Isoform level dynamics of AT4G36690 in sperm cells and vegetative nucleus.

**Supplemental Figure S9**. Isoform level dynamics of AT1G61140 in sperm cells and vegetative nucleus.

**Supplemental Figure S10**. Isoform level dynamics of AT3G01330 in sperm cells and vegetative nucleus.

**Supplemental Figure S11**. Isoform level dynamics of AT1G09140 in sperm cells and vegetative nucleus.

**Supplemental Figure S12**. Isoform level dynamics of AT3G58040 in sperm cells and vegetative nucleus.

**Supplemental Table S1**. Expression of genes in TPM level across 12 tissues.

**Supplemental Table S2**. Summary of datasets used for transcriptome assembly.

**Supplemental Table S3**. Quantification of genes undergoing alternative splicing in each cell type.

**Supplemental Table S4**. List of differentially expressed and spliced genes in the pairwise comparison between sperm cell and vegetative nucleus, and sperm cell and egg cell.

**Supplemental Table S5**. Gene Ontology (GO) analysis of differentially expressed and differentially spliced genes.

**Supplemental Table S6**. Alternative splicing events common to the three cell types.

**Supplemental Table S7**. List of isoform switch events of differentially alternatively spliced genes.

**Supplemental Table S8**. List of differentially expressed and spliced transcription factors and splicing factors.

**Supplemental Table S9**. List of primers used in this study.

## Supplementary Material

kiac574_Supplementary_Data
