# Supplementary material for: Cell-type-specific alternative splicing in the Arabidopsis germline
Source: Plant Physiol. 2022 Dec 14;192(1):85–101. doi: 10.1093/plphys/kiac574 (PMC10152659; doi:10.1093/plphys/kiac574)
Supplement: kiac574_Supplementary_Data [file kiac574_supplementary_data.zip › Supplemental Tables S2 S3 S9 and Figures S1S12.pdf]

Supplemental Tables and Figures

Supplemental Tables

Supplemental Table S2: Summary of datasets used for transcriptome assembly

Tissue	Total Reads	GEO Dataset	Reference
Root Hair	162253780	GSE85516	(Huang et al., 2017)
Root	129977077	GSE122772	(Tannenbaum et al., 2018)
Pollen	52181949	PRJNA194429	(Loraine et al., 2013)
Meiocyte	217130775	PRJNA342309	(Walker et al., 2017)
Egg cell	71896362	PRJNA495335	(Zhao et al., 2019)
Embryo	86389699	GSE121236	(Hofmann et al., 2019)
Leaf	77668303	E-MTAB-7978	(Mergner et al., 2020)
Seedlings	85669601	GSE109150	(Birkenbihl et al., 2018)
Silique	36049005	E-MTAB-7978	(Mergner et al., 2020)
Seed	41916346	E-MTAB-7978	(Mergner et al., 2020)
Sperm cell	107395784		This Study
Vegetative nucleus	101586723		This Study
**Total Reads**	1170115404		

Supplemental Table S3: Quantification of genes undergoing alternative splicing in each cell type

Splicing Summary	Sperm cell	Vegetative nucleus	Egg cell
Single-exon genes	921	1226	1613
Multi-exon genes	5859	7041	12041
Multi-transcript genes	2359	3332	6347
Number of genes spliced (%)	40.3	47.3	52.7

Supplemental Table S9: List of primers used in this study

**Primers**	**Sequence (5´→3´)**
AT1G61140_Fwd	ACGCTTTATCGGATTCTAGATGAGA
AT1G61140_Rev	TAAGCATCATCCCTGACACTAAAT
AT4G36690_Fwd	GCCCTAAATGGTATCAAGATGGG
AT4G36690_Rev	CAGGCTCCATAATCACCCTG
AT3G01330_Fwd	AAGATGCGGAATCGCTTGG
AT3G01330_Rev	AACCTAACCTCTCTCTCATCCC
AT1G09140_Fwd	CCAGTGGCCAGTTTTCATTT
AT1G09140_Rev	GTGTGAGTCGAAGCCCAGAT
AT3G58040_Fwd	GAATCCGAAGCAACGCATAATC
AT3G58040_Rev	CGCCTCGAAGTGTAAACAGAAC

Supplemental Figures

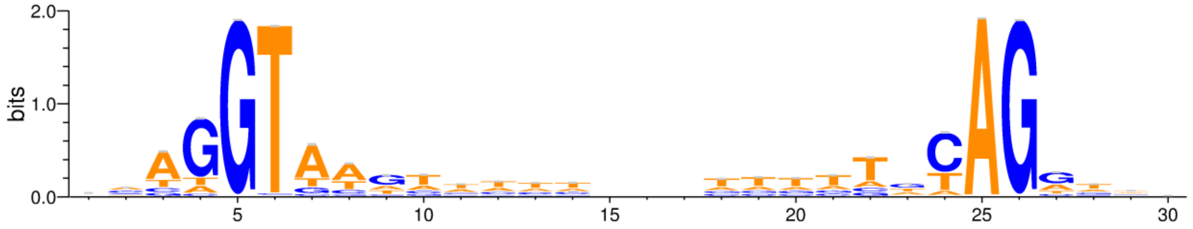

Supplemental Figure S1. Sequence logos of the exonic and the intronic boundaries of the 5´ and 3´ splice sites; logos were created using WebLogo (Crooks et al., 2004)**.**

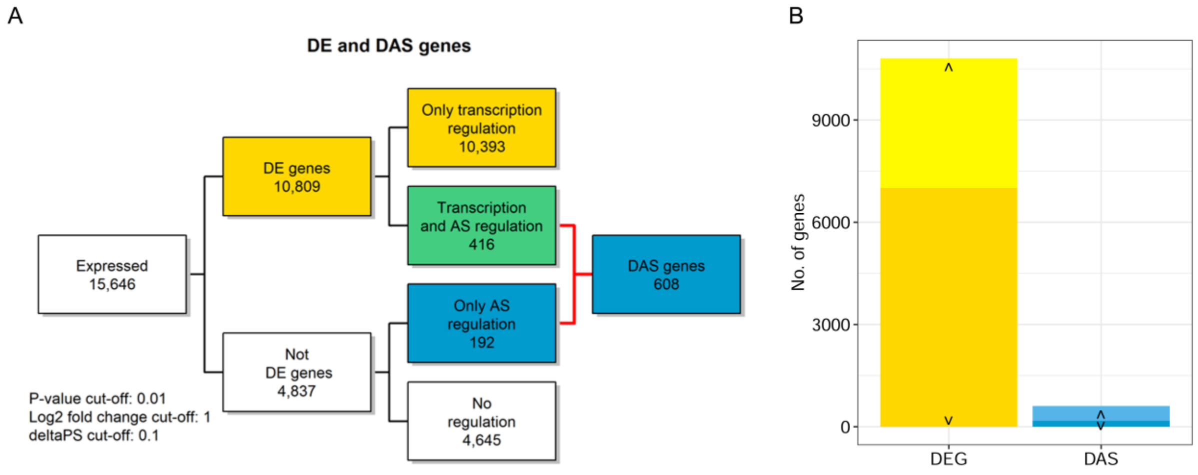

C

DEGs	GO term	GO ID	Count	FDR
UP	chromosome organization	GO:0051276	694	6.25E-33
	cell cycle	GO:0007049	690	7.00E-26
	DNA replication	GO:0006260	195	1.31E-22
	chromatin organization	GO:0006325	457	1.90E-16
	double-strand break repair	GO:0006302	137	1.64E-11
	nuclear division	GO:0000280	217	3.05E-10
	pollen sperm cell differentiation	GO:0048235	35	3.45E-10
	recombinational repair	GO:0000725	100	4.81E-10
	microgametogenesis	GO:0055046	58	1.55E-08
	nuclear chromosome segregation	GO:0098813	126	9.26E-08
	developmental cell growth	GO:0048588	220	1.07E-06
	male gamete generation	GO:0048232	66	4.59E-06
	histone modification	GO:0016570	236	0.000307
	regulation of protein modification process	GO:0031399	293	0.000694
	chromatin remodeling	GO:0006338	90	0.00107
DOWN	RNA processing	GO:0006396	993	1.16E-78
	peptide biosynthetic process	GO:0043043	865	4.49E-76
	translation	GO:0006412	860	1.72E-75
	ncRNA processing	GO:0034470	450	3.10E-70
	negative regulation of gene expression	GO:0010629	375	1.13E-22
	gene silencing	GO:0016458	217	7.37E-18
	embryo development	GO:0009790	439	1.04E-05
	fruit development	GO:0010154	657	2.1E-05
	protein processing	GO:0016485	59	0.045572

D

	GO term	GO ID	Count	FDR
DAS	RNA processing	GO:0006396	993	3.34E-09
	RNA splicing	GO:0008380	357	3.06E-07
	chromosome organization	GO:0051276	694	5.17E-07
	posttranscriptional regulation of gene expression	GO:0010608	325	4.53E-05
	negative regulation of gene expression	GO:0010629	375	0.000214
	chromatin organization	GO:0006325	457	0.003173
	DNA-dependent DNA replication	GO:0006261	148	0.009069

Supplemental Figure S2: Differential expression analysis at gene and transcript level between sperm cell and egg cell. A) Flowchart depicting analysis of gene expression and alternative splicing between sperm cells and egg cell. Numbers of differentially expressed (DE) genes and differentially alternatively spliced (DAS) genes in sperm cell vs vegetative nucleus comparison. **B)** Bar plots showing the number of genes that are differentially expressed (DEG) and differentially alternatively spliced (DAS), separated by upregulated (^) and downregulated (v). **C)** Functional enrichment of upregulated and downregulated differentially expressed genes between sperm and egg cell. **D)** Functional enrichment of differentially spliced genes between sperm and egg cell. For details see Supplemental Table S5.

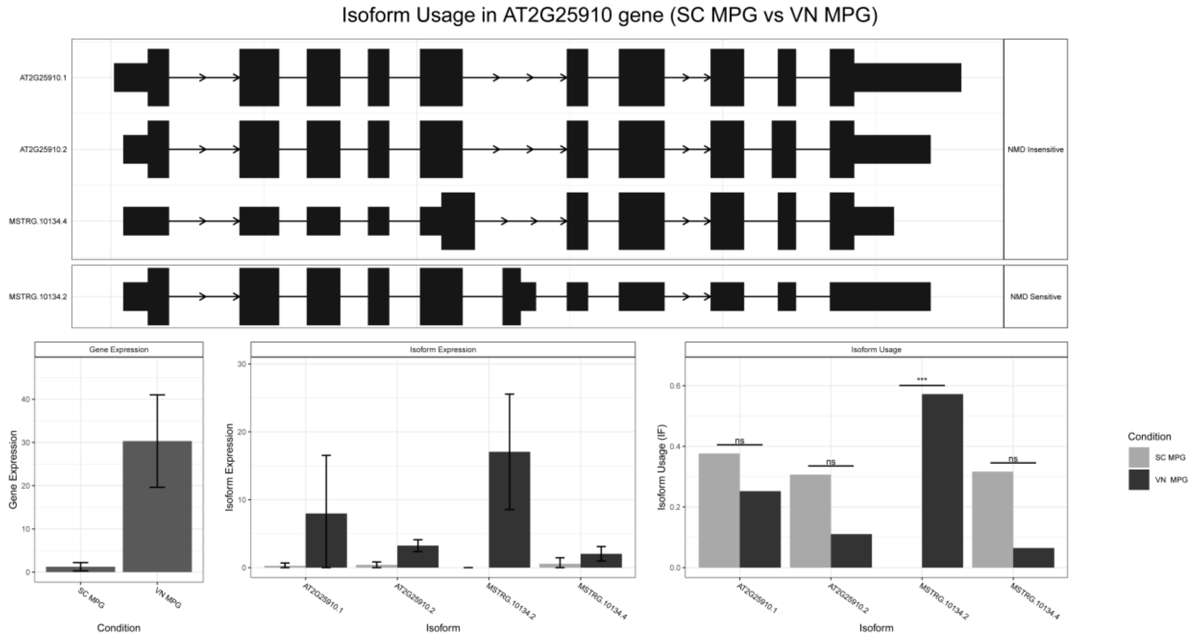

Supplemental Figure S3: **Overview of the number of isoforms predicted to have functional consequences in AT2G25910 analysed by IsoformSwitchAnalyzeR** (Vitting-Seerup and Sandelin, 2019). The upper panel shows the gene models and the potential consequences at RNA level (NMD). The lower panel shows the expression of isoform and their usage in sperm cell vs vegetative nuclei comparison. The error bar indicates the standard error (SE) across 3 biological replicates (n = 3). SC sperm cell, VN vegetative nucleus, MPG mature pollen grain, NMD nonsense-mediated mRNA decay, ns non-significant.

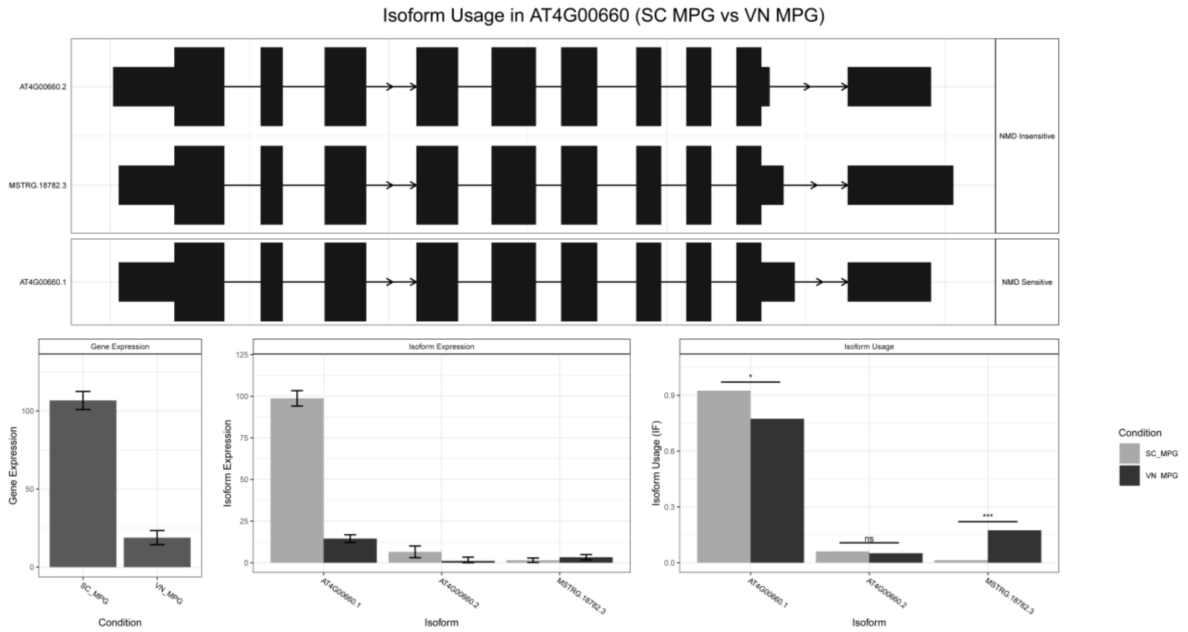

Supplemental Figure S4: **Overview of the number of isoforms predicted to have functional consequences in AT4G00660 analysed by IsoformSwitchAnalyzeR** (Vitting-Seerup and Sandelin, 2019). The upper panel shows the gene models and the potential consequences at RNA level (NMD). The lower panel shows the expression of isoform and their usage in sperm cell vs vegetative nuclei comparison. The error bar indicates the standard error (SE) across 3 biological replicates (n = 3). SC sperm cell, VN vegetative nucleus, MPG mature pollen grain, NMD nonsense-mediated mRNA decay, ns non-significant.

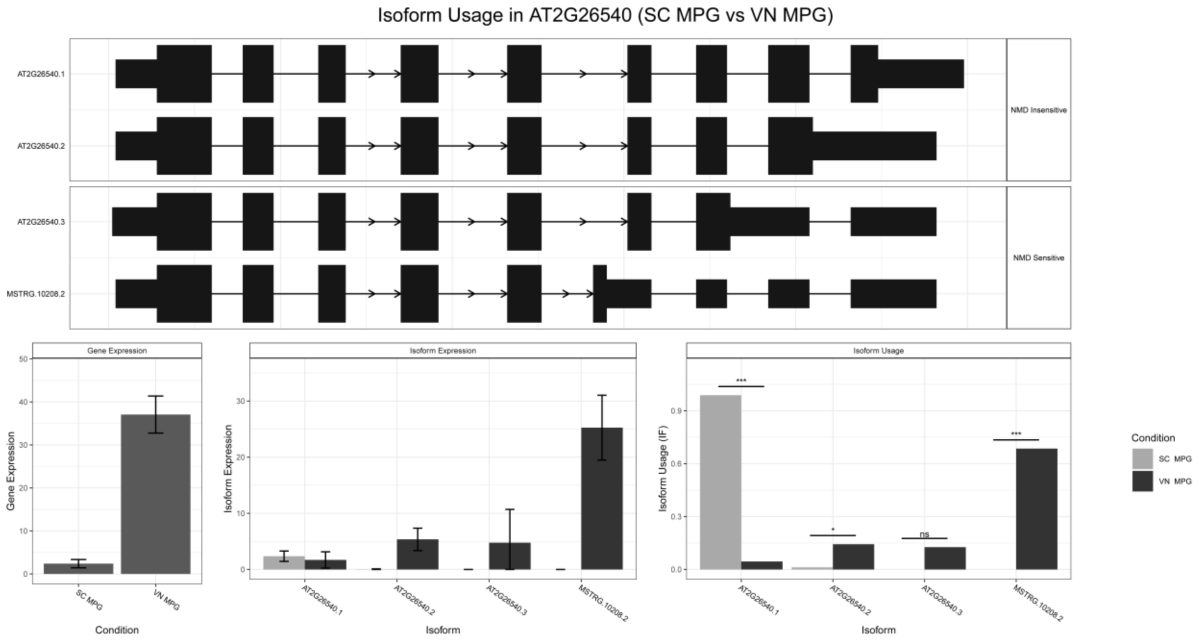

Supplemental Figure S5: Overview of the number of isoforms predicted to have functional consequences in AT2G26540 analysed by IsoformSwitchAnalyzeR (Vitting-Seerup and Sandelin, 2019). The upper panel shows the gene models and the potential consequences at RNA level (NMD). The lower panel shows the expression of isoform and their usage in sperm cell vs vegetative nuclei comparison. The error bar indicates the standard error (SE) across 3 biological replicates (n = 3). SC sperm cell, VN vegetative nucleus, MPG mature pollen grain, NMD nonsense-mediated mRNA decay, ns non-significant.

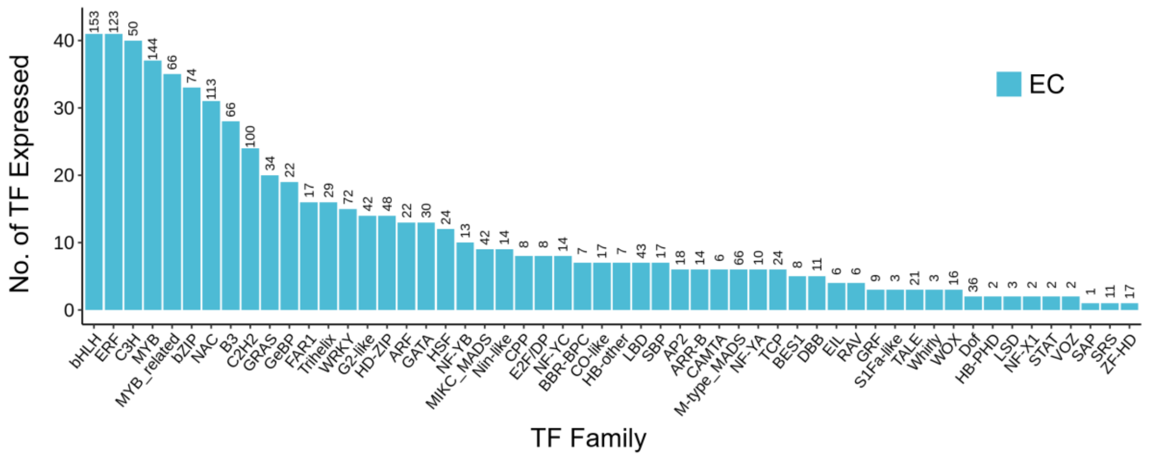

Supplemental Figure S6: **Number of transcription factors (TFs) per family expressed in egg cell (EC).**

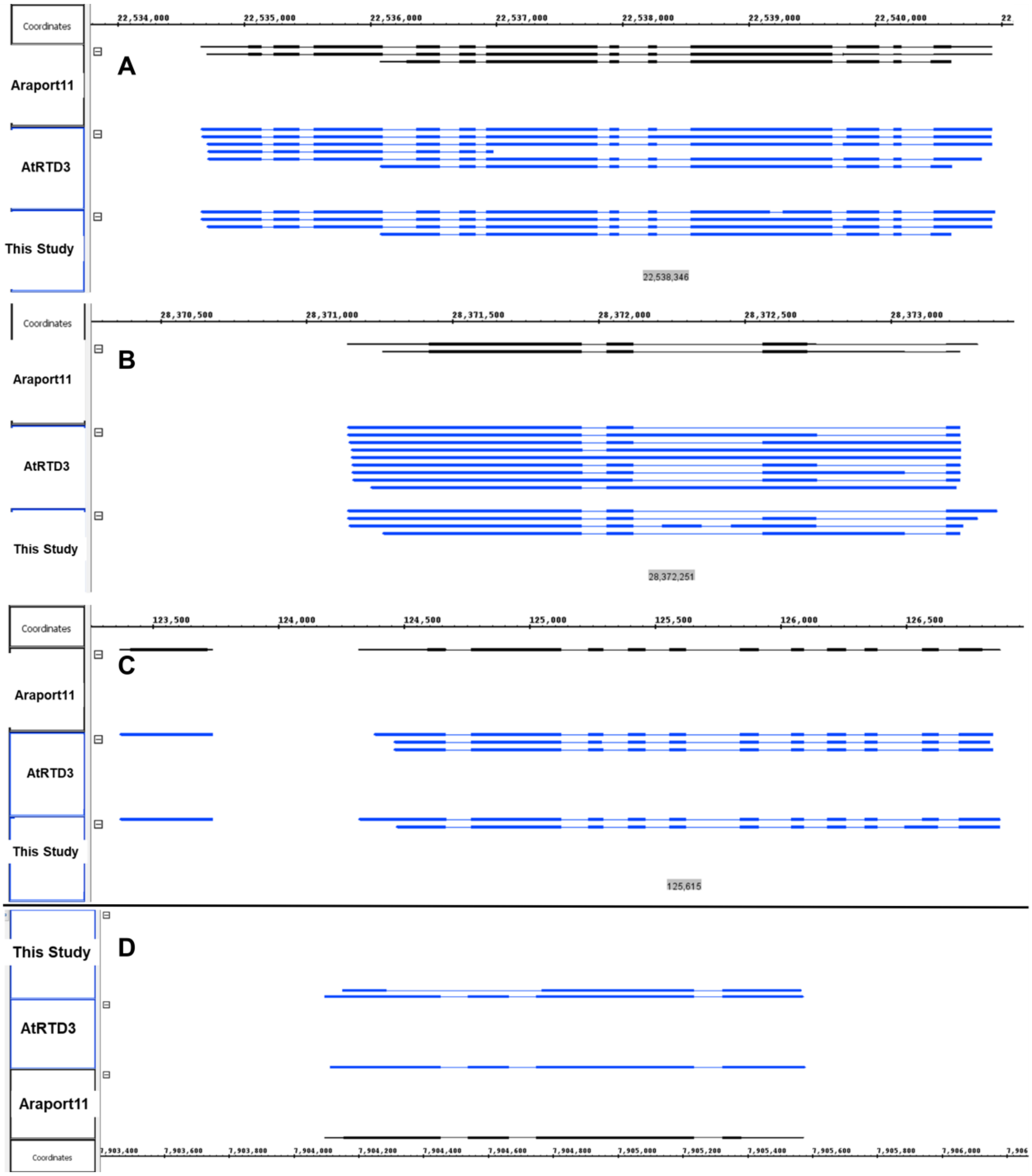

Supplemental Figure S7: Visualization of predicted gene models on IGB (Freese et al., 2016) for comparison between Araport11, AtRTD3 and this study for four representative genes: AT1G61140 (A), AT1G75560 (B), AT3G01330 (C), AT3G22360 (D).

.

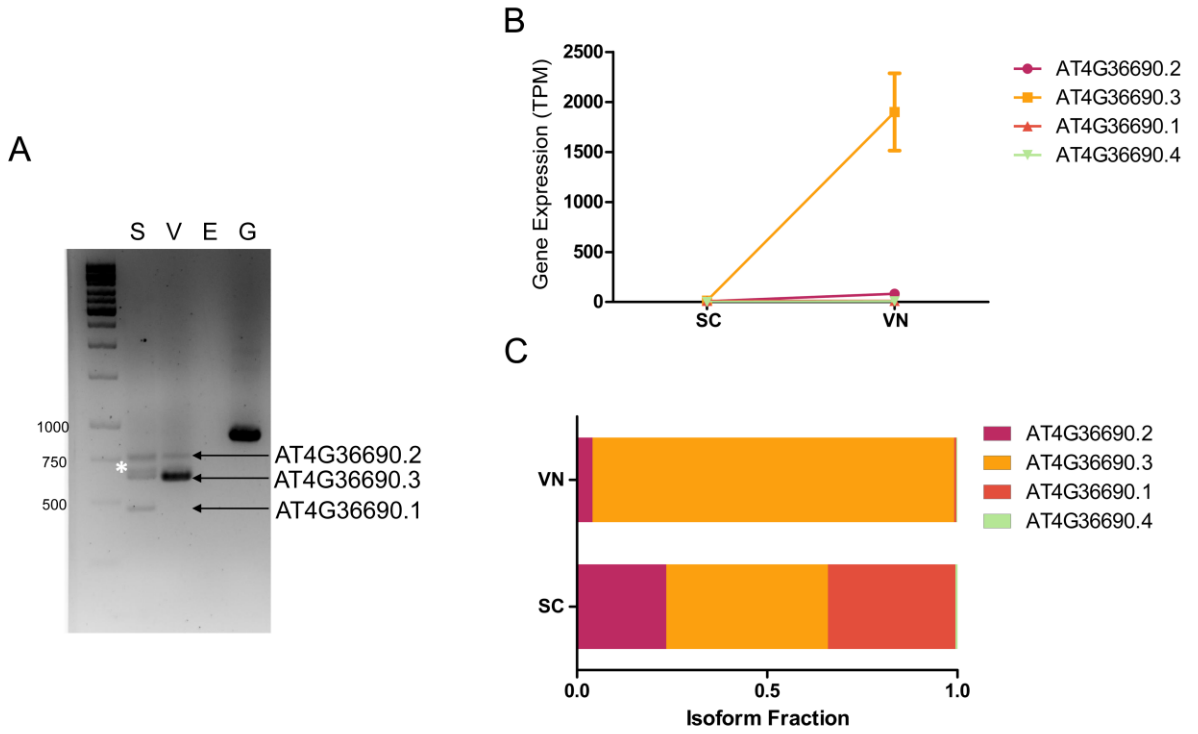

Supplemental Figure S8: Isoform level dynamics of AT4G36690 in sperm cells and vegetative nucleus. A) RT-PCR followed by gel electrophoresis confirmation of male germline specific spliced isoforms. Arrows indicate different isoforms that were identified by RT-PCR. Isoform AT4G36690.1 (467bp) appears in sperm while not in vegetative nucleus. AT4G36690.2 (740bp) appears in both sperm cell and vegetative nucleus, though with less intensity in vegetative nucleus. AT4G36690.3 (640bp) appears as a strong band in vegetative nucleus. AT4G36690.4 could not be resolved by RT-PCR, probably due to low expression. One additional unpredicted band (~650bp) marked with white asterisks was detected in the sperm cell sample and might represent an isoform not predicted by our RNA-seq pipeline or unspecific amplification, respectively. **B)** Expression level (TPM) of individual isoforms that are differentially expressed between sperm cells and vegetative nucleus. **C)** Changes in the isoform fraction of the corresponding gene shown in B. S sperm cell, V vegetative nucleus, E empty well, G genomic DNA, SC sperm cell, VN vegetative nucleus, TPM Transcripts per million. The error bar indicates standard deviation across 3 biological replicates ($n = 3$).

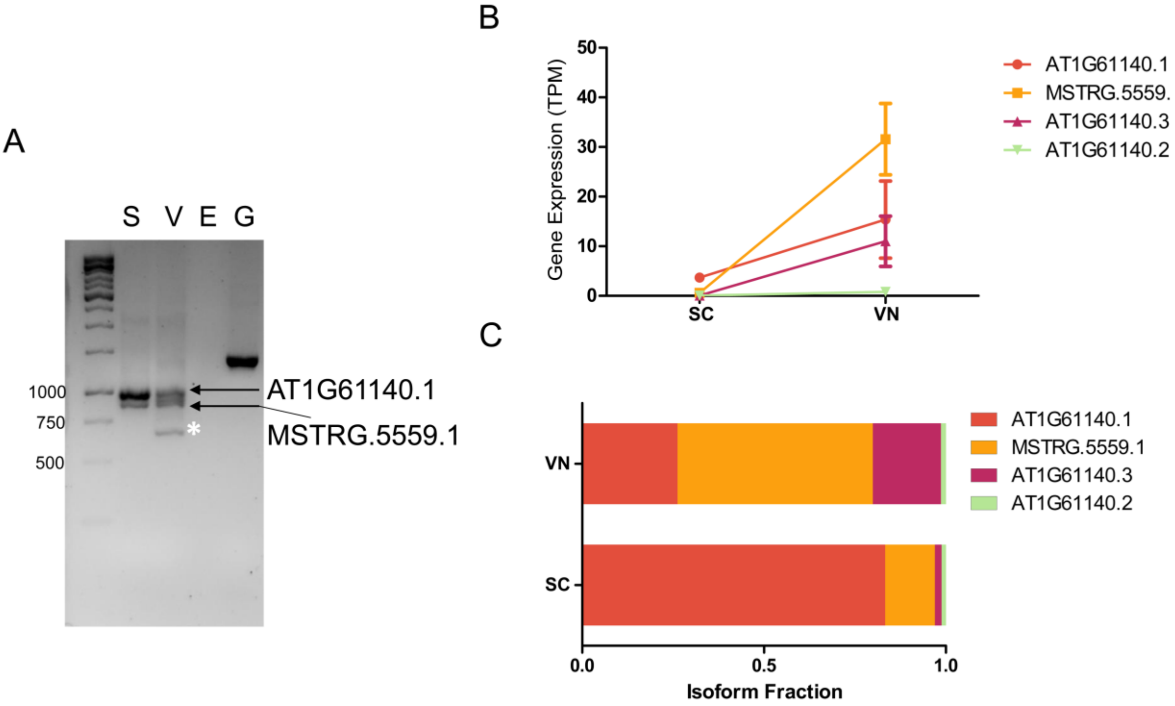

Supplemental Figure S9: Isoform level dynamics of AT1G61140 in sperm cells and vegetative nucleus. A) RT-PCR followed by gel electrophoresis confirmation of male germline specific spliced isoforms. Arrows indicate different isoforms that were identified by RT-PCR. AT1G61140.1 (977bp) is shown in both sperm cell and vegetative nucleus. MSTRG.5559.1 (877bp) showed expression in both sperm and vegetative nucleus. The unpredicted band (~650bp) indicated with a white asterisk was found in vegetative nucleus but could not be identified by our RNA-seq pipeline. We could not detect AT1G61140.3 (1004bp) probably due to similar size of AT1G61140.1. White asterisk indicates potential new transcripts not identified by our AS splicing pipeline. **B)** Expression level (TPM) of individual isoforms that are differentially expressed between sperm cells and vegetative nucleus. **C)** Changes in the isoform fraction of the corresponding gene show in B. S sperm cell, V vegetative nucleus, E empty well, G genomic DNA, SC sperm cell, VN vegetative nucleus, TPM Transcripts per million. The error bar indicates standard deviation across 3 biological replicates ($n = 3$).

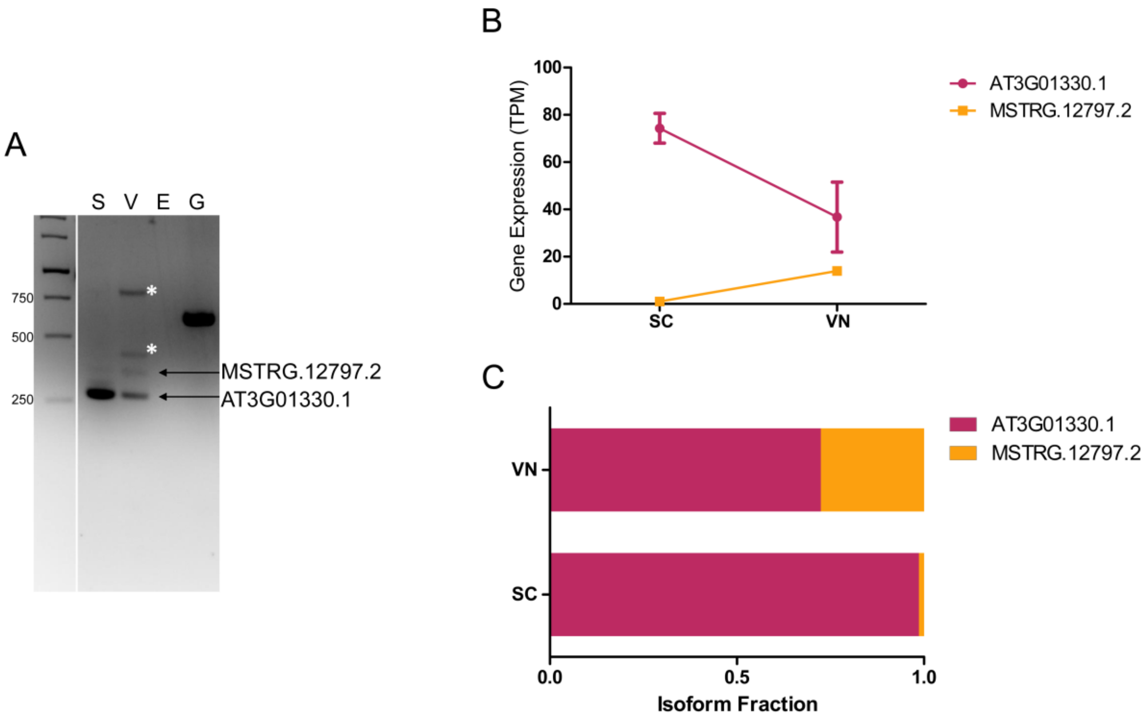

Supplemental Figure S10: Isoform level dynamics of AT3G01330 in sperm cells and vegetative nucleus. A) RT-PCR followed by gel electrophoresis confirmation of male germline specific spliced isoforms. Arrows indicate different isoforms that were identified by RT-PCR. AT3G01330.1 (287bp) was found to be expressed in sperm and vegetative nucleus, while an additional predicted band indicating isoform MSTRG.12797.2 (366bp) was found to be expressed in vegetative nucleus. Two additional unpredicted bands (~480bp and 900bp) marked with white asterisks were detected in the vegetative nucleus sample and might represent an isoform not predicted by our RNA-seq pipeline or unspecific amplification, respectively. **B)** Expression level (TPM) of individual isoforms that are differentially expressed between sperm cells and vegetative nucleus. **C)** Changes in the isoform fraction of the corresponding gene show in B. S sperm cell, V vegetative nucleus, E empty well, G genomic DNA, SC sperm cell, VN vegetative nucleus, TPM Transcripts per million. The error bar indicates standard deviation across 3 biological replicates ($n = 3$).

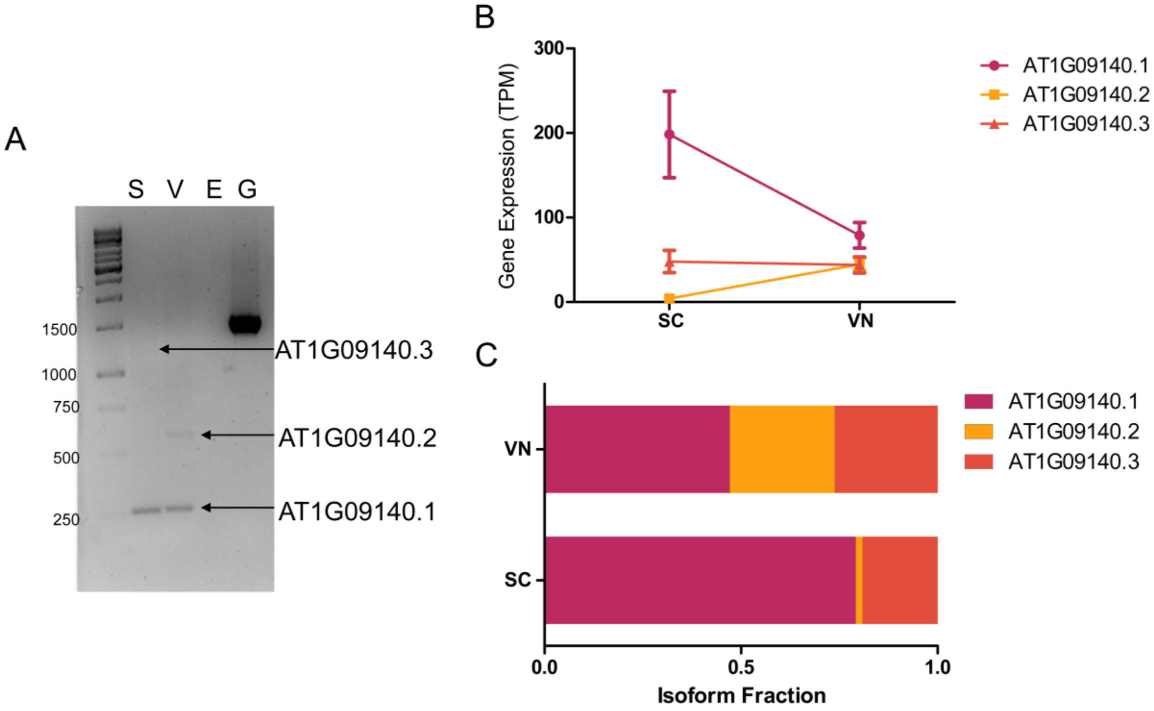

Supplemental Figure S11: Isoform level dynamics of AT1G09140 in sperm cells and vegetative nucleus. A) RT-PCR followed by gel electrophoresis confirmation of male germline specific spliced isoforms. Arrows indicate different isoforms that were identified by RT-PCR. AT1G09140.2 (601bp) was identified and found to be expressed in vegetative nucleus. Isoform AT1G09140.1 (262bp) was found to be expressed in both sperm cell and vegetative nucleus. We could not detect AT1G09140.3 (1204bp) in vegetative nucleus but it was faintly detected in sperm cell. **B)** Expression level (TPM) of individual isoforms that are differentially expressed between sperm cells and vegetative nucleus. **C)** Changes in the isoform fraction of the corresponding gene show in B. S sperm cell, V vegetative nucleus, E empty well, G genomic DNA, SC sperm cell, VN vegetative nucleus, TPM Transcripts per million. The error bar indicates standard deviation across 3 biological replicates ($n = 3$).

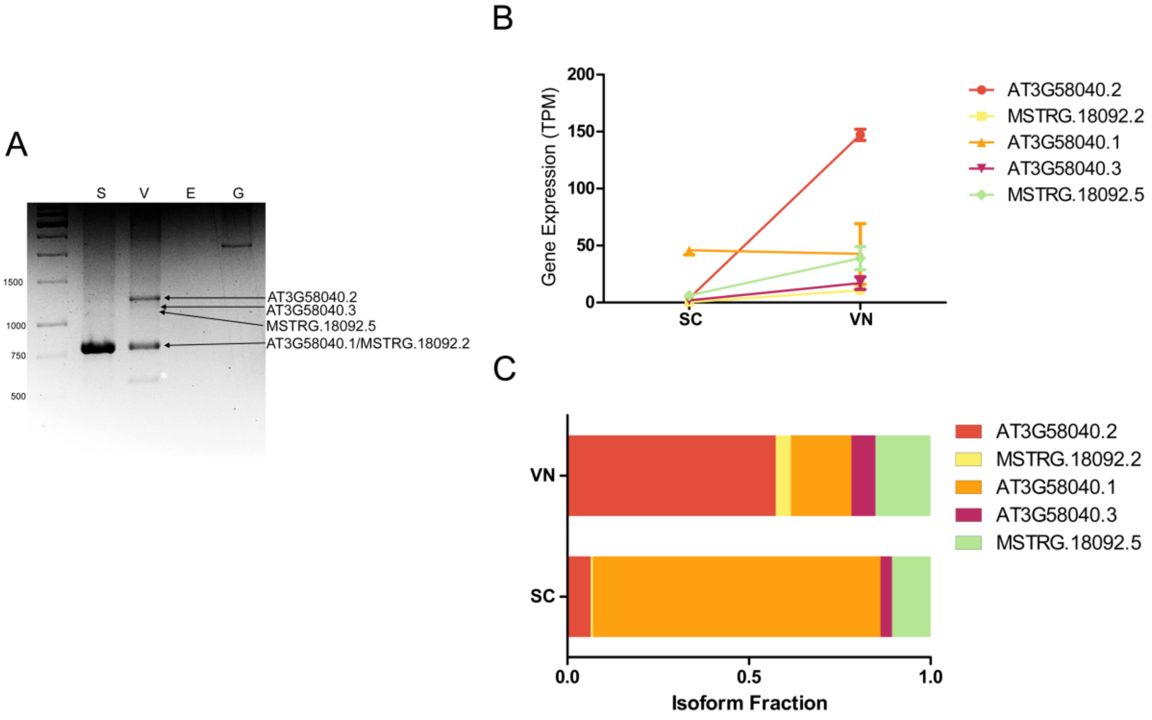

Supplemental Figure S12: Isoform level dynamics of AT3G58040 in sperm cells and vegetative nucleus. A) RT-PCR followed by gel electrophoresis confirmation of male germline specific spliced isoforms. Arrows indicate different isoforms that were identified by RT-PCR. AT3G58040.2 (1259bp) was identified and found to be expressed in vegetative nucleus. Isoform AT3G58040.3 (~1188bp) and MSTRG.18092.5 (~1180bp) was found to be faintly expressed in vegetative nucleus. Isoform AT3G58040.1 (~793bp) was found to be highly expressed sperm cells and in vegetative nucleus. Isoform MSTRG.18092.2 (~810bp) seems to be overlapped with the bands of isoform AT3G58040.1. One additional unpredicted band (~600bp) marked with white asterisk was detected faintly in the sperm cell and relatively higher expressed in the vegetative nucleus sample and might represent an isoform not predicted by our RNA-seq pipeline or unspecific amplification, respectively. **B)** Expression level (TPM) of individual isoforms that are differentially expressed between sperm cells and vegetative nucleus. **C)** Changes in the isoform fraction of the corresponding gene show in B. S sperm cell, V vegetative nucleus, E empty well, G genomic DNA, SC sperm cell, VN vegetative nucleus, TPM Transcripts per million. The error bar indicates standard deviation across 3 biological replicates ($n = 3$).

References

Birkenbihl RP, Kracher B, Ross A, Kramer K, Finkemeier I, Somssich IE (2018) Principles and characteristics of the Arabidopsis WRKY regulatory network during early MAMP-triggered immunity. Plant J **96**: 487–502

Crooks GE, Hon G, Chandonia J-M, Brenner SE (2004) WebLogo: a sequence logo generator. Genome Res **14**: 1188–1190

Freese NH, Norris DC, Loraine AE (2016) Integrated genome browser: visual analytics platform for genomics. Bioinformatics **32**: 2089–2095

Hofmann F, Schon MA, Nodine MD (2019) The embryonic transcriptome of Arabidopsis thaliana. Plant Reprod **32**: 77–91

Huang L, Shi X, Wang W, Ryu KH, Schiefelbein J (2017) Diversification of Root Hair Development Genes in Vascular Plants. Plant Physiol **174**: 1697–1712

Loraine AE, McCormick S, Estrada A, Patel K, Qin P (2013) RNA-seq of Arabidopsis pollen uncovers novel transcription and alternative splicing. Plant Physiol **162**: 1092–1109

Mergner J, Frejno M, List M, Papacek M, Chen X, Chaudhary A, Samaras P, Richter S, Shikata H, Messerer M, et al (2020) Mass-spectrometry-based draft of the Arabidopsis proteome. Nature **579**: 409–414

Tannenbaum M, Sarusi-Portuguez A, Krispil R, Schwartz M, Loza O, Benichou JIC, Mosquna A, Hakim O (2018) Regulatory chromatin landscape in Arabidopsis thaliana roots uncovered by coupling INTACT and ATAC-seq. Plant Methods **14**: 113

Vitting-Seerup K, Sandelin A (2019) IsoformSwitchAnalyzeR: analysis of changes in genome-wide patterns of alternative splicing and its functional consequences. Bioinformatics **35**: 4469–4471

Walker J, Gao H, Zhang J, Aldridge B, Vickers M, Higgins JD, Feng X (2017) Sexual-lineage-specific DNA methylation regulates meiosis in Arabidopsis. Nat Genet. doi: 10.1038/s41588-017-0008-5

Zhao P, Zhou X, Shen K, Liu Z, Cheng T, Liu D, Cheng Y, Peng X, Sun M-X (2019) Two-Step Maternal-to-Zygotic Transition with Two-Phase Parental Genome Contributions. Dev Cell **49**: 882-893.e5
